# Isomaltooligosaccharides Sustain the Growth of *Prevotella* Both *In Vitro* and in Animal Models

**DOI:** 10.1128/spectrum.02621-21

**Published:** 2022-11-15

**Authors:** Junkui Chen, Zhengpeng Li, Xiaofan Wang, Bin Fan, Feilong Deng, Hongwei D.Yu, Xiaolei Ze, Liying Zhu, Yeshi Yin, Yanhong Chen, Jiangchao Zhao, Yunsheng Yang, Xin Wang

**Affiliations:** a State Key Laboratory for Managing Biotic and Chemical Threats to the Quality and Safety of Agro-products, Institute of Food Science, Zhejiang Academy of Agricultural Sciences, Hangzhou, People’s Republic of China; b Department of Gastroenterology and Hepatology, Chinese PLA General Hospital, Beijing, People’s Republic of China; c Department of Animal Science, Division of Agriculture, University of Arkansas, Fayetteville, Arkansas, USA; d Department of Critical Care Medicine, Zhejiang Provincial People’s Hospital, People’s Hospital of Hangzhou Medical College, Hangzhou, People’s Republic of China; e Department of Biomedical Sciences, Marshall University, Huntington, West Virginia, USA; f BYHEALTH CO., Ltd., Guangzhou, People’s Republic of China; g Hunan University of Science and Engineering, Yongzhou, Hunan, People’s Republic of China; h Laboratory Animal Center of Zhejiang University, Hangzhou, People’s Republic of China; University of Michigan-Ann Arbor

**Keywords:** *Prevotella*, isomalto-oligosaccharides, microbiome, short chain fatty acids

## Abstract

The human digestive tract is colonized by trillions of bacterial cells that play important roles in human health and diseases. It is well known that dietary habits are associated with human microbiota enterotypes. However, the factors that determine the enterotype still remain elusive. In this study, it was first examined, via *in vitro* batch fermentation, how different carbohydrates affect the *Bacteroides* and *Prevotella* enterotypes. Among the 11 substrates (fructo-, galacto-, xylo-, manno-, and isomalto-oligosaccharides [IMO] and lactulose, raffinose, starch, inulin [INU], mannitol, and xylitol) tested, IMO, INU, and starch were found to sustain the growth of *Prevotella* through batch fermentation. The development of the *Prevotella* and *Bacteroides* enterotypes was further simulated in chemostats using fecal samples. IMO coupled with faster dilution rates and lower pH were required to sustain the growth of Prevotella copri in the chemostat based on 16S rRNA gene and metagenomic sequencing. Meanwhile, starch with relatively lower dilution rates and higher pH was required to support the development of the *Bacteroides* enterotype. Amylo-α-1,6-glucosidase, pectin, and xylan lyases were the carbohydrate-active enzymes associated with the *Prevotella* enterotype. The *Bacteroides* enterotype was associated with more diversified carbohydrate-active enzymes. Consistently, since honey contains high isomaltose content, mice fed IMO and honey displayed an increased relative abundance of *Prevotella* in the colon. In conclusion, both *in vitro* systems and a mouse model were used to demonstrate that IMO maintains the *Prevotella* enterotype. This result provides insight into the nutritional requirements underlying gut enterotype formation.

**IMPORTANCE** The *Prevotella* enterotype type is a human traditional enterotype with high dietary fiber intake, which is related to healthy ageing and Parkinson’s disease development. Manipulations of the dwelled gut microbes by dietary isomalto-oligosaccharides efficiently sustained *Prevotella* type enterotypes, indicating that it can be used in the improvement of elderly health by increasing the gut transit time.

## INTRODUCTION

The human digestive tract is colonized by trillions of bacterial cells that influence the host’s health status through multiple host-bacterium interactions (1). Environmental factors, such as household, diet, and lifestyle, are major driving forces that shape the structure of the human gut microbiota (2). This leads to the idea that the unique characteristics of host-specific microbiota affect the efficacy of oral medical and nutritional interventions. Food components with diverse chemical and physical properties help to determine microbial assemblages in the human gut, and this is not a random process (3). The modulation of the colonic microbiota by defined dietary components, such as microbiota-accessible carbohydrates (MACs) or microbiota-directed foods (MDFs) (4), can help with prophylactic treatment of human illness, such as type II diabetics, metabolic syndrome, and obesity (5). MACs, designated by Sonnenburg, refer to a range of oligo- and polysaccharides that can be utilized by the human colonic microbiome (6). Meanwhile, MDFs are food components that can be used to selectively manipulate the gut microbiota to benefit host health (4). The chemical properties of mono-sugar units, linkage bonds, and chain lengths are the major factors that influence the degree of degradation in the distal intestine by the gut microbiota. This, in turn, determines the composition and function of colonic microbiota.

The human gut microbiome can be classified into three distinct clusters, which are designated *Bacteroides* (B-), *Prevotella* (P-), and *Ruminococcus* enterotypes (7). The factors that determine the enterotype composition remain unclear, although a link between dietary habits and at least two enterotypes has been repeatedly confirmed (8). Higher protein and fat intake is associated with the B-enterotype, while higher fiber intake, particularly xylan and pectin intake, is linked to the P-enterotype. A recent publication by Tett et al. reported that a Westernized lifestyle has been associated with the reduction of *P. copri* prevalence (9). Genome analysis of various *Prevotella* species clusters has revealed the enrichment of xylan catabolism enzymes (10). However, a previous publication by Wu et al. suggested that dietary glucose was highly associated with the prevalence of *Prevotella* (11). Considering that the microbiota associated with the B- and P-enterotypes may possess unique carbohydrate-active enzymes, the utilization profiles of MACs may differ between the B- and P-enterotypes. In other words, MACs with different chemical structures and molecular weights may support the development of different enterotypes. Given that bacterial enterotypes mediate the relationship between dietary habits and health abnormalities (12), understanding how dietary components, such as MACs, selectively enhance the growth of specific groups of gut microbiota is of particular interest.

In this study, the growth of human fecal microbiota and short chain fatty acid (SCFA) production was first examined in response to a range of MACs in an *in vitro* batch fermentation system. This analysis revealed that the individualized fecal microbiota displayed heterogeneous responses to the MACs. Further, isomalto-oligosaccharides (IMO) were identified as a key group of nutrients supporting the growth of *Prevotella in vitro*. Finally, the stimulatory effects of IMO and IMO-enriched food (honey) on Prevotella copri growth were validated using a mouse model.

## RESULTS

### Gut microbiome responses to different MACs in *in vitro* batch culture.

The raw sequences obtained from batch fermentation were processed using QIIME2. This resulted in a total of 8,053,584 reads. The mean of reads was 41,513 (±1,100 standard deviations [SD]), with the number of reads per sample ranging from 10,508 to 86,717. These sequences were assigned to bacterial operational taxonomic units (OTUs) with a 1-nucleotide variation. The OTUs were classified into 9 bacterial phyla, 70 families, 248 genera, and 446 species.

**FIG 1:**
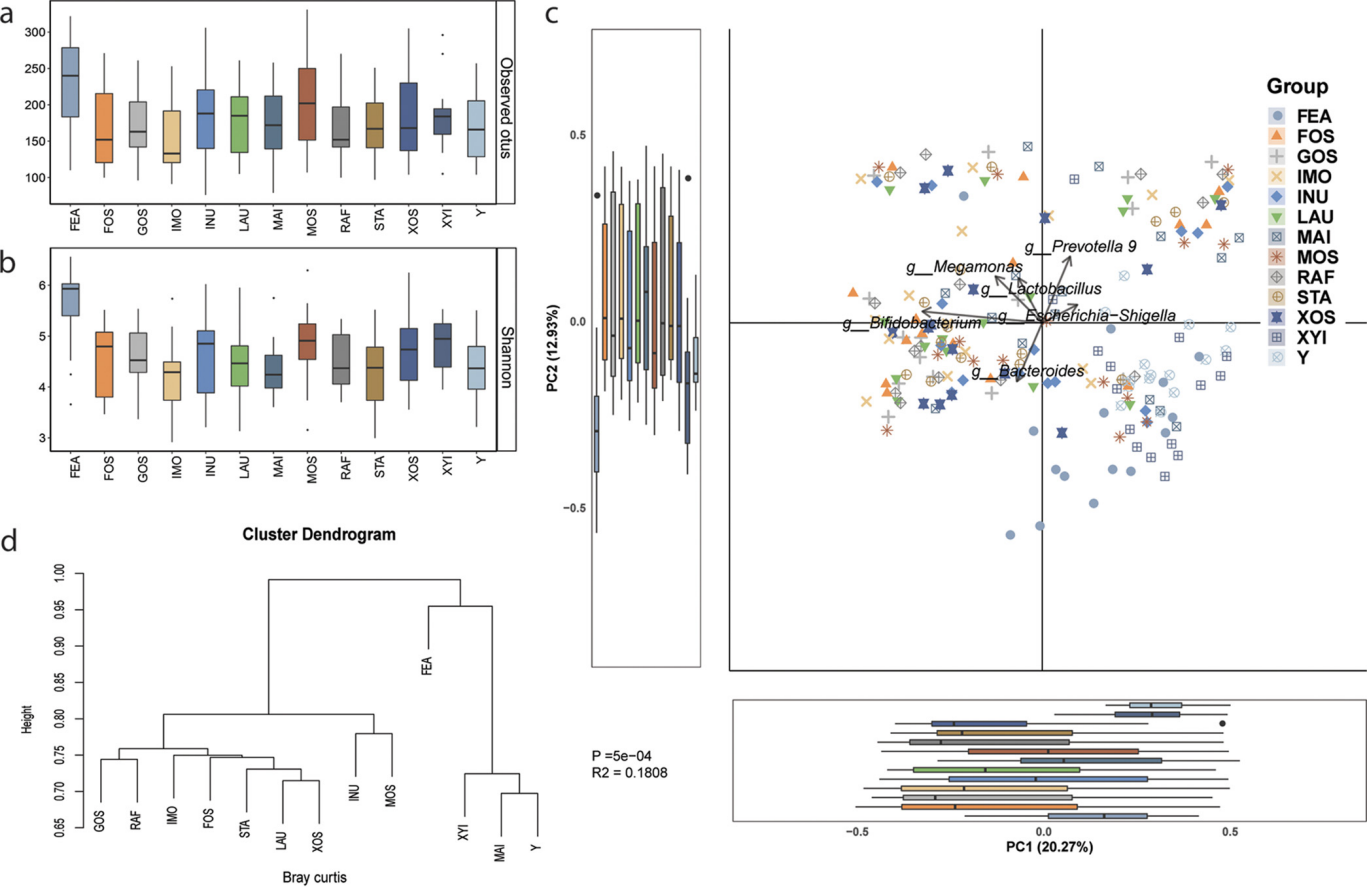
(a to d) Responses of gut microbiota to tested microbiota-accessible carbohydrates (MACs) in *in vitro* batch fermentation cultures: (a) observed OTUs; (b) Shannon index; (c) principal-component analysis; (d) (cluster dendrogram analysis). The substrates tested were fructooligosaccharides (FOS), galactooligosaccharides (GOS), isomaltooligosaccharides (IMO), inulin (INU), lactulose (LAU), mannitol (MAI), mannooligosaccharides (MOS), raffinose (RAF), starch (STA), xylooligosaccharides (XOS), xylitol (XYI), and control medium (Y). Fecal slurry microbiotas are displayed as FEA.

An average of 175 OTUs, with a range of 76 to 331 OTUs, were observed in the batch culture bottles. In general, *in vitro* batch fermentation led to reduced microbial richness with respect to the original fecal samples (175 and 225 observed OTUs, respectively; Fig. 1a). Among the tested carbohydrates, IMO generated the lowest OTU numbers (Fig. 1a). A similar pattern was also observed for the overall microbial richness and evenness (Shannon index; Fig. 1b). Supplementation with mannooligosaccharides (MOS) resulted in the highest number of bacterial OTUs (*n* = 204) and bacterial diversity (culturing time of 48 h) among all the MACs.

The impacts of the tested MACs on the compositions of the microbiota after fermentation were analyzed by principal-component analysis (PCA). *Bifidobacterium* and Escherichia*-Shigella* were the two major genera ascribed to the PCA1 clustering, while *Bacteroides* and *Prevotella 9* were the major factors that contributed to the PCA2 clustering (Fig. 1c). The clustering of samples in the PCA figure can be more clearly visualized in the clustering dendrogram (Fig. 1d). One cluster included the microbiota communities after IMO, xylooligosaccharide (XOS), raffinose (RAF), fructooligosaccharide (FOS), galactooligosaccharide (GOS), lactulose (LAU), and starch (STA) fermentation; another cluster constituted the microbiota communities after inulin (INU), mannitol (MAI), and MOS fermentation, and the third cluster included those after fermentation in control (Y), fecal microbiota (FEA), and xylitol (XYI) media.

The impacts of different carbohydrates on the *in vitro* batch cultures of the gut microbiota were illustrated at the phylum level (Fig. S1). Among the total phyla, *Firmicutes* was the most abundant phylum in the original feces (78.5%). The relative abundance of *Firmicutes* declined in all batch culture media supplemented with MACs (38.6% relative abundance on average). Fermentation in XYI-supplemented growth medium yielded the best growth of *Firmicutes*, with an average abundance of 55.9%. *Bacteroidetes*, *Proteobacteria*, and *Actinobacteria* were also dominant phyla, representing 9.0%, 7.2%, and 4.6% of the total phyla, respectively, in the original fecal samples. Their growth was found to be promoted by certain MACs. Supplementing the media with oligosaccharides (such as MOS and GOS) facilitated better growth of *Bacteroidetes* (19.3% and 17.8% relative abundance, respectively) compared to sugar alcohol groups, such as XYI (10.4%). A similar trend was also observed for *Actinobacteria*. The relative abundance of *Actinobacteria* was greatly enriched after GOS- or IMO-supplemented fermentation but not in the media containing XYI, and MAI. For *Proteobacteria*, the culture without any carbohydrates (Y) led to the highest relative abundance (53.9%). Meanwhile, the presence of FOS, GOS, and RAF resulted in the lowest *Proteobacteria* abundances of 15.9%, 13.7%, and 14.5%, respectively.

The next step was to analyze the effect of MACs on the gut microbiome at the genus level by examining the correlations between MACs and individual genera (Fig. S2). In general, the abundances of *Kluyvera*, *Sutterella*, *Enterococcus*, *Allisonella*, and Escherichia*-Shigella* were higher in all tested media than in the original feces. Meanwhile, genera including *Faecalibacterium*, *Roseburia*, *Paraprevotella*, and *Ruminococcus* were decreased under all culture conditions. Of note, the overall bacterial profiles in XYI were similar to those in the YCFA medium (Y; described in Material and Methods). Increased growth of *Bifidobacterium* was detected in all media except the XYI and Y groups.

### Clustering the microbiome in original fecal samples.

**FIG 2:**
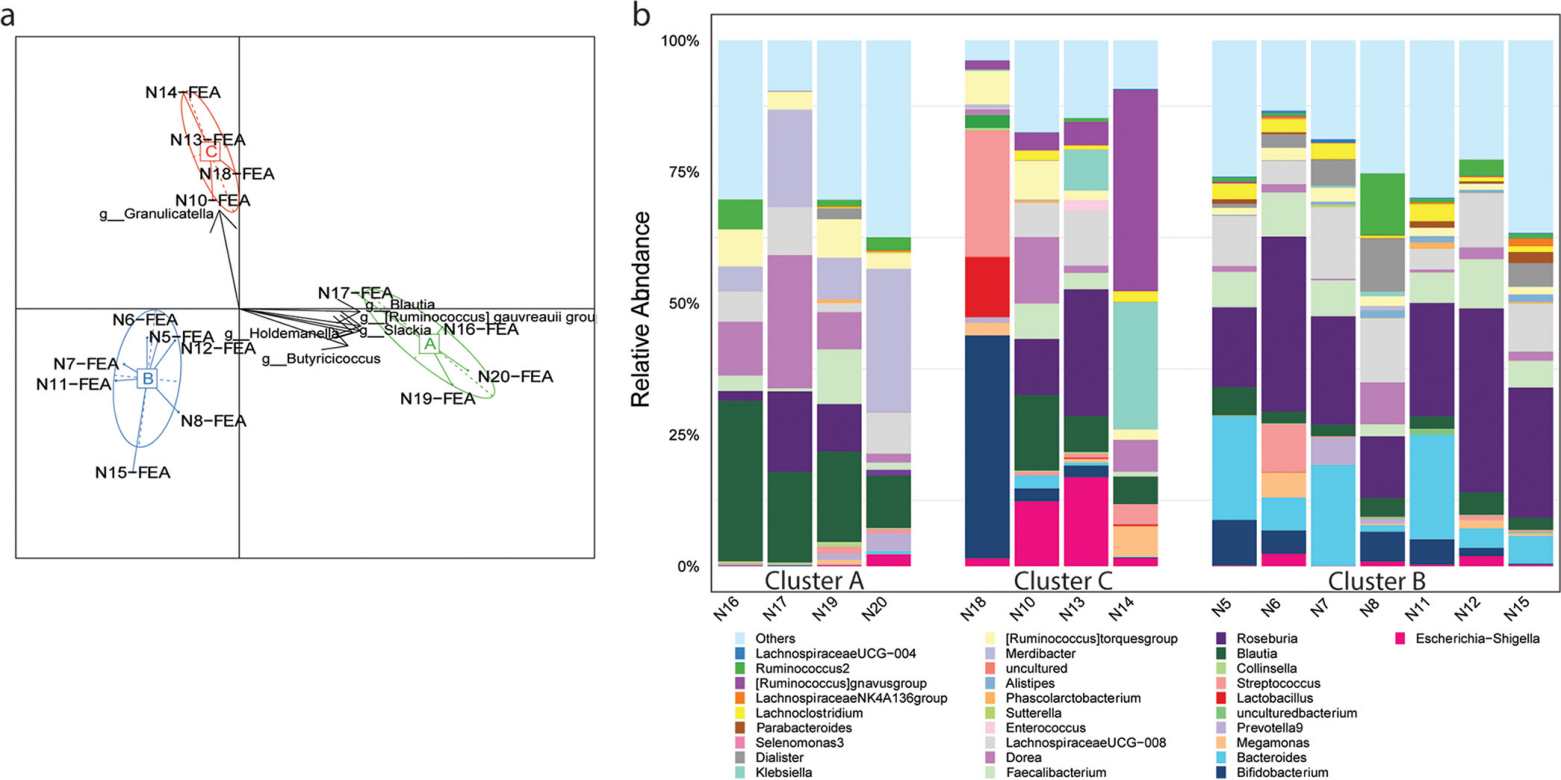
The gut microbiome community structures of 15 human volunteers based on analysis of fecal samples. (a) Principal-component analysis (PCA) of the fecal microbiota (FEA) compositions of 15 volunteers. (b) Relative abundances of bacterial genera in the three human gut microbial community types (clusters A, B, and C) detected via 16S rRNA sequencing.

The 15 volunteers were classified according to their gut microbiome community structures, which were clearly separated into three distinguishable clusters on the PCA plot (Fig. 2a). Interestingly, feces from volunteers belong to cluster A, including N16, N17, N19, and N20 were enriched in *Prevotella* and lower in *Bifidobacterium* abundance (Fig. 2b). Random forest analysis of feces in cluster A revealed the enrichment of the *Lachnospiraceae* FCS020 group, *Ruminiclostridium* 5, and *Clostridium sensu stricto-1* (Fig. S3). Cluster B-type fecal samples, from volunteers N5, N6, N7, N8, N11, N12, and N15, were high in *Bacteroides*. Random forest analysis enabled the identification of signature genera, such as *Parabacteroides*, *Bacteroides*, and *Alistipes* in the feces of this cluster. Feces from volunteers N10, N13, N14, and N18 were clustered into cluster C. Cluster C feces were high in *Granulicatella*, *Lactobacillus*, and *Turicibacter* (Fig. 2 and Fig. S3).

### Substrates selectively boost the growth of *Prevotella* and *Bacteroides*.

**FIG 3:**
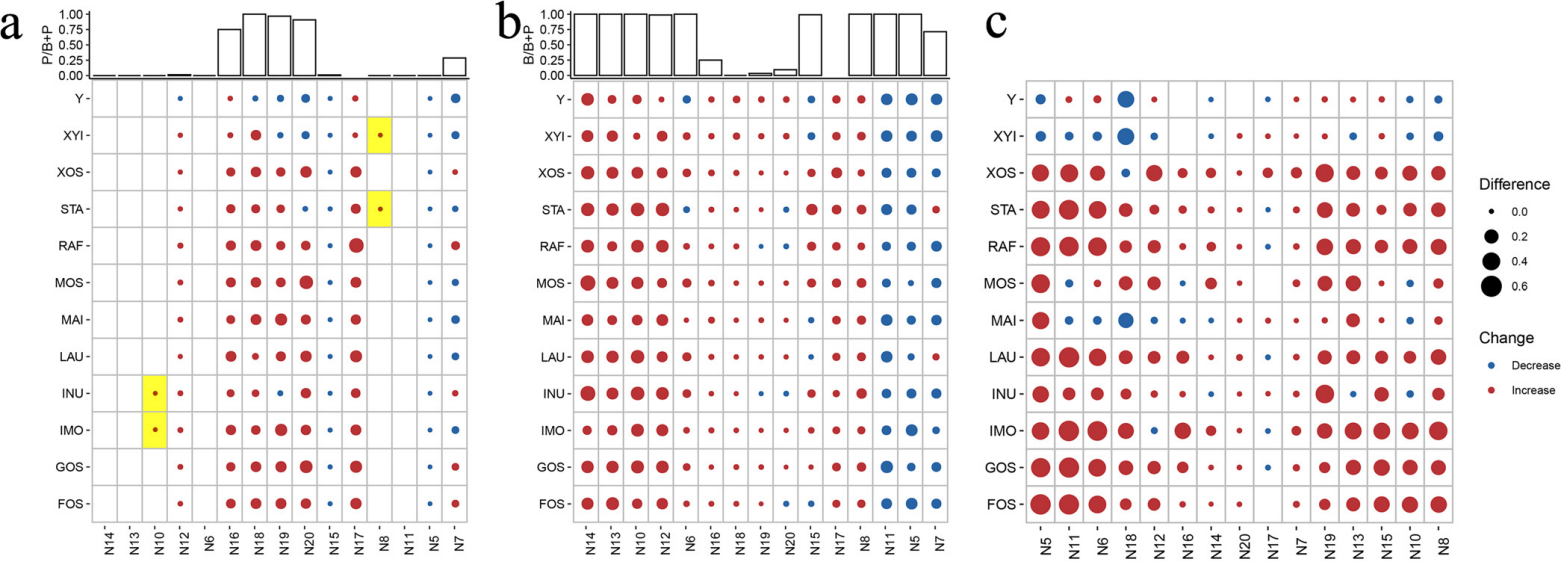
Responses of the microbiota from 15 human fecal samples to growth substrates. (a to c) The effects of the tested substrates on the growth of (a) *Prevotella* (P), (b) *Bacteroides* (B), and (c) *Bifidobacterium* are shown. The red and blue dots represent the increases and decreases in bacterial abundance, respectively. The yellow boxes indicate that the population increased from undetectable levels to detectable levels in the feces after fermentation. The substrates tested were fructooligosaccharides (FOS), galactooligosaccharides (GOS), isomaltooligosaccharides (IMO), inulin (INU), lactulose (LAU), mannitol (MAI), mannooligosaccharides (MOS), and raffinose (RAF).

The next step was to analyze the effects of the tested substrates on the growth of *Prevotella* and *Bacteroides*. Higher growth rates of *Prevotella* were detected in the media containing XOS, FOS, GOS, LAU, MOS, IMO, RAF, and MAI. Meanwhile, the growth rates of *Prevotella* were relatively lower in XYI and YCFA media (Fig. 3a). Interestingly, increased growth of *Prevotella* was consistently detected in the cultures that used samples from cluster A (N16, N17, N19, and N20) as the inoculum (Fig. 3a). The different substrates tended to have very limited effects on the growth of *Bacteroides*. Significant changes in the growth rates of *Bacteroides* were detected in fecal samples with a higher proportion of *Bacteroides* among the total *Bacteroidaceae* (*Bacteroides* divided by the sum of *Prevotella* and *Bacteroides*; Fig. 3b).

The enrichment of *Bifidobacterium* was affected by both fecal enterotypes and substrates. Increased growth of *Bifidobacterium* was detected in the cultures under a range of supplements, including XOS, STA, RAF, GOS, and FOS. However, the enrichment was limited in the N20, N17, N7, N14, and N16 fecal samples (Fig. 3c).

Batch fermentation revealed successful growth of *Prevotella* in the growth media supplemented with most MACs when using fecal samples with high *Prevotella* abundance. It is worth noting that IMO, INU, XYI, and STA promoted *Prevotella* abundance, even in the cultures with low abundances of *Prevotella* (Fig. 2a). Hence, these substrates may serve as essential nutrient sources for *Prevotella* growth.

### Association of microbiota composition with SCFA production in response to MACs.

The concentrations of SCFAs, including acetate, propionate, butyrate, isobutyrate, valerate, and isovalerate, were measured after 48 h of fermentation (13). In general, the acetate and propionate concentrations increased in all MAC-supplemented media after 48 h of cultivation. Interestingly, among all groups, MAI-supplemented fermentation generated the largest amount of propionate but the smallest amount of acetate. Butyrate production was reduced significantly after culturing in growth media supplemented with FOS, GOS, IMO, RAF, LAU, STA, and XOS but increased significantly in the XYI medium (Fig. S4).

Spearman’s rank correlation test (false-discovery rate [FDR], <0.1) was used to determine the positive and negative associations between the tested substrates and bacterial community members (Fig. S5). The genera that were positively correlated with acetate production included *Tyzzerella 3*, *Ventriosum* group, *Agathobacter*, *Barnesiella*, *Lachnospira*, *Lachnospiraceae_Other*, *Roseburia*, and *Faecalibacterium.* Meanwhile, several genera, such as *Stackia*, *Prevotella 9*, *Senegalimassilia*, and *Holdemanella*, were negatively associated with acetate production (Fig. S5a). *Megamonas* was the main bacterial genus that was associated with propionate production in all substrate groups. Other genera, including *Erysipelatoclostridium*, *Allisonella*, and Klebsiella, were positively associated with propionate production in more than one substrate group (Fig. S5b). *Sutterella* was mainly associated with butyrate production (Fig. S5c). There was a negative association found between butyrate production and *Bifidobacterium*.

### IMO stimulated the growth of *Prevotella* in chemostat systems.

Although IMO, INU, XYI, and STA promoted the growth of low abundances of *Prevotella* in the culture based on the results of *in vitro* batch fermentation, the growth rate was found to be relatively lower in XYI medium compared to IMO, and the latter demonstrated the higher growth rate for *Prevotella*. Moreover, a previous study in our group found that soluble starch was a good substrate to sustain the growth of *Bacteroides* in single-stage chemostat regardless of the enterotypes (14). Hence, inulin-type substrate was considered another candidate for the chemostat experiment. Since FOS has the same chemical structure as inulin but a similar degree of polymerization as IMO, and has shown the ability to promote the growth of *Prevotella*, FOS and IMO were then applied further in a single-stage chemostat system.

**FIG 4:**
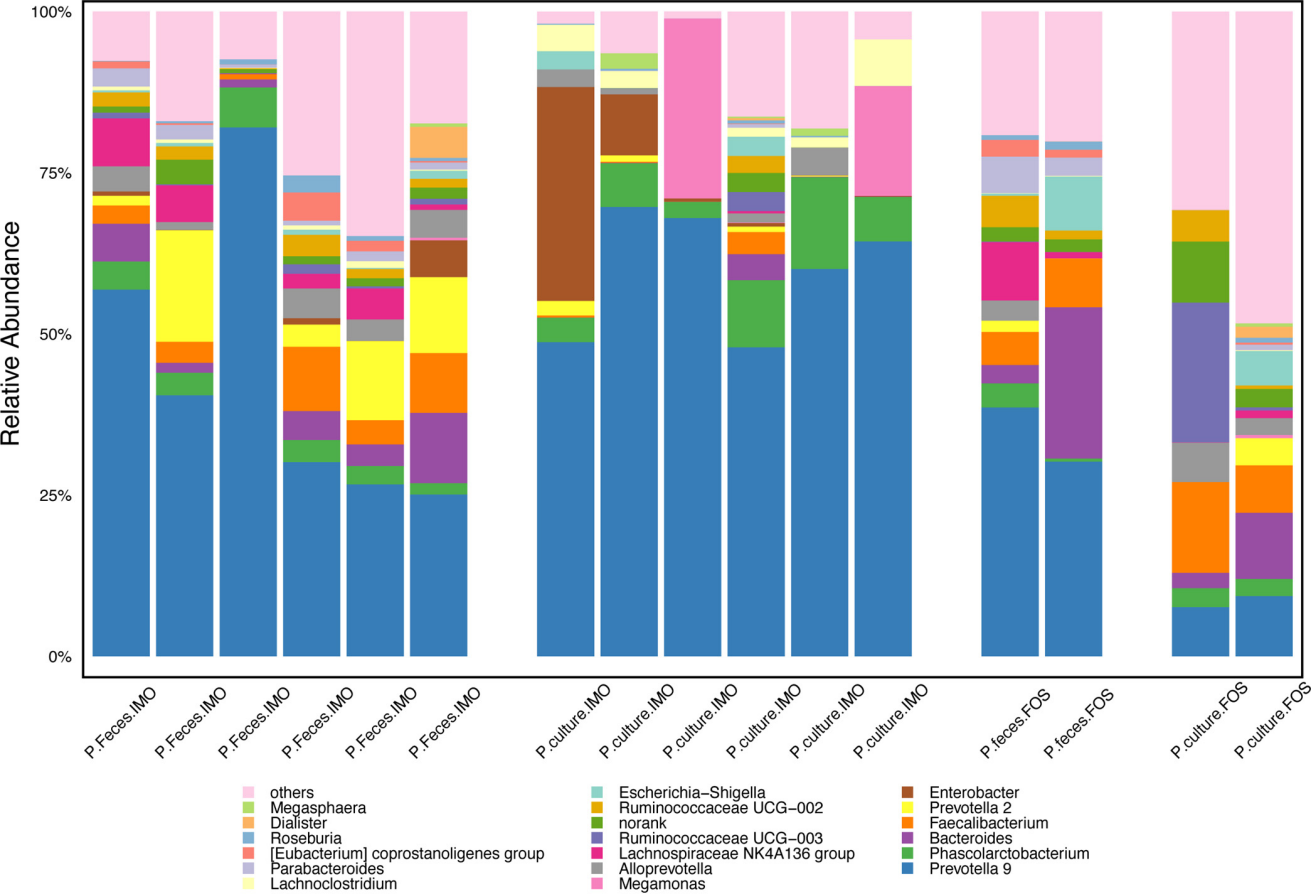
Gut microbial community composition in original fecal samples (before culturing) and chemostat culture products (after culturing). Eight human fecal samples containing *Prevotella* were cultured in chemostat systems in the presence of isomaltooligosaccharides (IMO) or fructooligosaccharides (FOS) for 11 days. The relative abundances of bacterial genera were detected via 16S rRNA gene sequencing.

At the beginning of experiments, we tested the impacts of dilution rates and pH on the abundance of *Prevotella* in the chemostats. However, we found that the population of *Prevotella* cannot been enhanced in four parallel chemostat systems containing IMO and controlled at pH 6.5 with dilution rates of 0.083/h, 0.042/h, 0.027/h, and 0.021/h (Fig. S6). In subsequences experiments, we found that low pH in combination with the fastest dilution rate (0.083/h) sustained the growth of *Prevotella* in the chemostat at steady stage (Fig. S7). Then six fecal samples with higher *Prevotella* abundance were individually inoculated into the chemostats containing IMO and FOS to further verify the growth conditions required by *Prevotella*. As presented in Fig. 4, *Prevotella* was observed in all chemostat cultures, so both the IMO- and FOS-supplemented media supported the growth of *Prevotella* (Fig. 4). The IMO culture elevated the relative abundance of *Prevotella* by 16% (from 52% to 60%). However, culturing with FOS caused a 66% reduction (from 35% to 11%) in *Prevotella* abundance. Instead, the abundances of *Lactobacillus* and *Ruminococcaceae* UCG-014 were promoted by FOS to over 15% from less than 1%. Using the chemostat culture system with IMO supplementation, a highly sustainable *Prevotella*-dominated microbial community was simulated.

### Metagenome analysis of communities from the chemostat culture systems.

According to previous reports, *Bacteroides*, *Prevotella*, and *Ruminococcus* have been identified as the three enterotypes that are common to all human gut microbiomes, and they are influenced by long-term dietary regimes (15). Therefore, the next stage of the present study was to investigate the metagenomic and functional changes in the gut microbiota after culturing in chemostats with different substrates and enterotype origins. Four B-enterotype feces samples were cultured in chemostats with soluble starch with a dilution rate of 0.042/h and pH of 6.2. Six P-enterotype feces samples were cultured with IMO with a dilution rate of 0.08/h and a pH of 5.5. Samples collected before and after the chemostat culturing were subjected to DNA extraction and shotgun sequencing.

**FIG 5:**
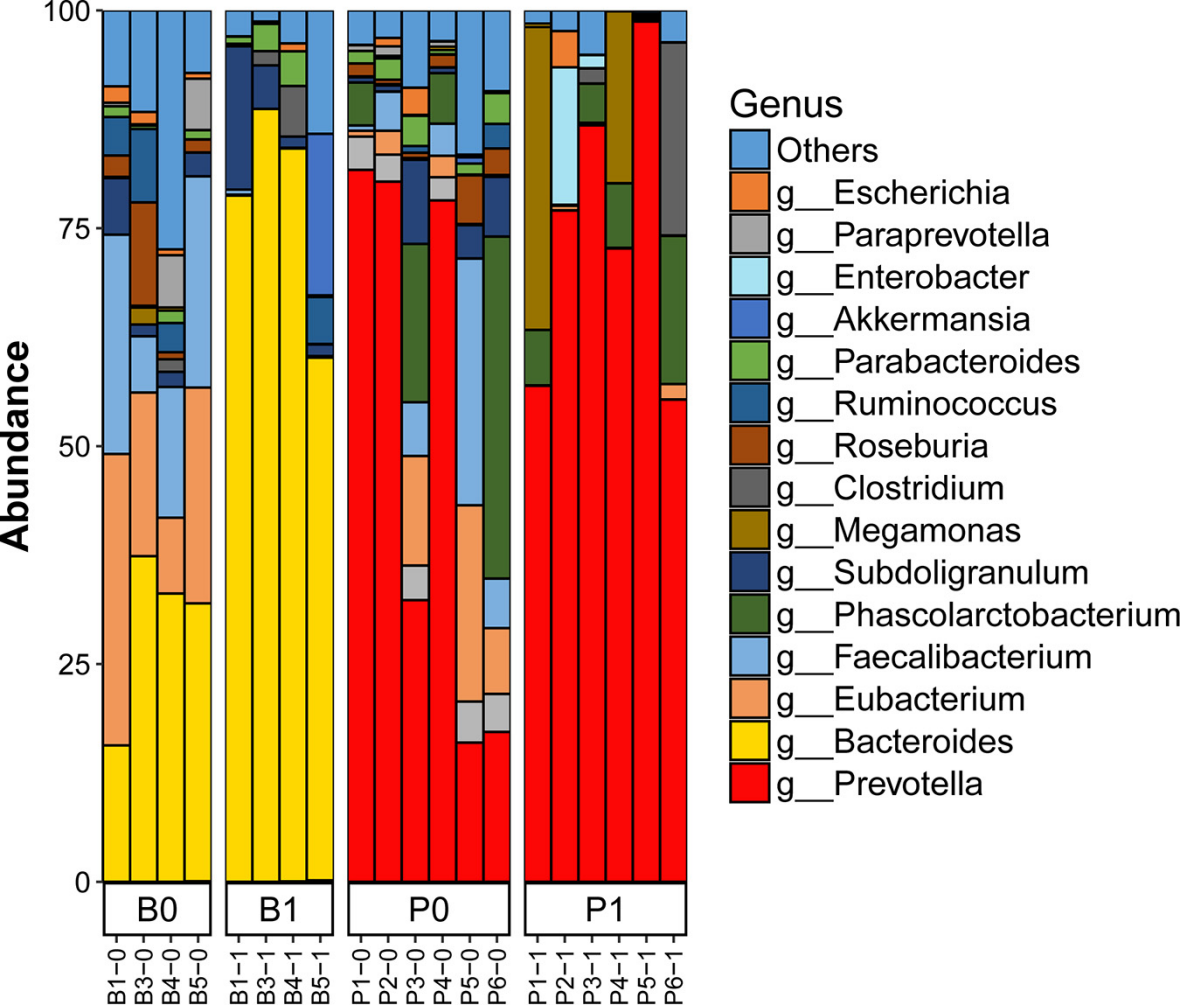
Bacterial community composition at the species level in the original fecal samples and chemostat culture products inoculated with *Prevotella* (P) and *Bacteroides* (B) enterotype fecal slurries as detected by metagenomics. B1-0 to B5-0 and P1-0 to P6-0 represent the original fecal samples, and B1-1 to B5-1 and P1-1 to P6-1 represent the corresponding chemostat products.

A total of 513,113,972 raw paired-end reads (approximately 154 Gb) were obtained from the 20 samples. After removing duplications, reads from human sources, and low-quality reads, an average of 7.55 Gb of high-quality sequence data was obtained for each sample. These data were assembled to an average of 45,134 contigs per sample using SoapDenovo2.0. A total of 1,162,411 nonredundant genes were obtained from all 20 samples using MetaGeneMarker (version 1). A total of 321,930 genes matched genes in the NCBI-nucleotide (NT) database (downloaded on 12 May 2017). Of these genes, 288,879 (identity, >85%) and 201,119 (identity, >95%) were assigned at the genus and species levels, respectively. After reconstructing the shotgun sequence data for each sample, *Bacteroides* and *Prevotella* were the predominant genera detected in the chemostat cultures containing soluble starch and IMO, respectively, as the sole carbon sources (Fig. 5). At the species level, Bacteroides uniformis was the predominant species maintained at high abundance in the chemostat cultures inoculated with the four individual B-enterotype feces. In addition, Bacteroides thetaiotaomicron was detected in three chemostat cultures. A total of 22 *Bacteroides* species, including B. coprocola, B. vulgatus, B. fragilis, and B. stercoris, were detected in the human feces. However, these species did not grow well in the chemostats using the fermentation parameters adopted in this study (Fig. S8). Prevotella copri was the only *Prevotella* species maintained alive at the steady-state level in the cultures involving all six fecal inocula with IMO as the sole carbon source. Other *Prevotella* species, including P. stercorea, P. disiens, and P. bivia, were only detected in the chemostat cultures of one or two of the fecal samples. In contrast to the total of 22 *Bacteroides* species identified, only 4 *Prevotella* species were found in the feces.

**FIG 6:**
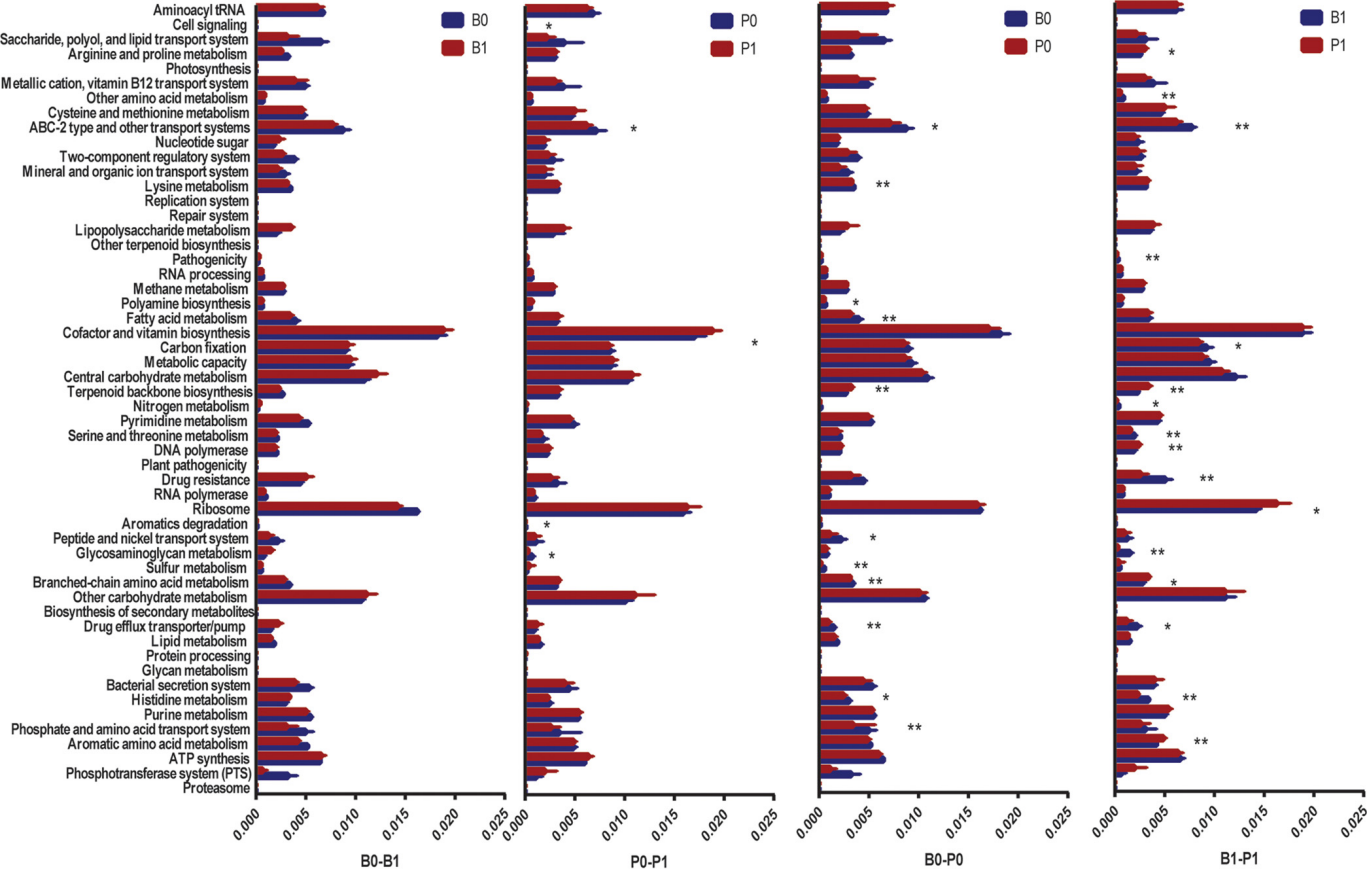
Kyoto Encyclopedia of Genes and Genomes (KEGG) annotations associated with *Bacteroides* (B-) and *Prevotella* (P-) enterotypes in the original fecal samples (B0 and P0 before culturing) and after chemostat simulation (B1 and P1). The abundance of KEGG module level c annotations before and after the chemostat simulation of the two enterotypes based on the four fecal samples collected from *Bacteroides* (B-) enterotypes and six collected from *Prevotella* (P-) enterotypes. The Wilcoxon *P* value was used to test the differences in abundance. *, *P* < 0.05; **, *P* < 0.01.

A total of 481,368 (42.1%) and 58,225 (5%) genes matched those in the KEGG and Carbohydrate-Active enZYmes (CAZy) databases, respectively. After assignment to the KEGG catalogs, the fecal samples of the two enterotypes and their corresponding chemostat culture products significantly differed with respect to the top 25 most abundant catalogs. The B-enterotype samples, from both feces and chemostat cultures, were enriched in the pathways of histidine metabolism, drug efflux transporter/pump, ABC-2 type and other transport systems, and sulfur metabolism. In contrast, the P-enterotype samples were enriched in the pathway of terpenoid backbone biosynthesis (Fig. 6). Moreover, there were no significant differences in the pathway analysis between the feces and chemostat culture products for either the B- or P-enterotype.

**FIG 7:**
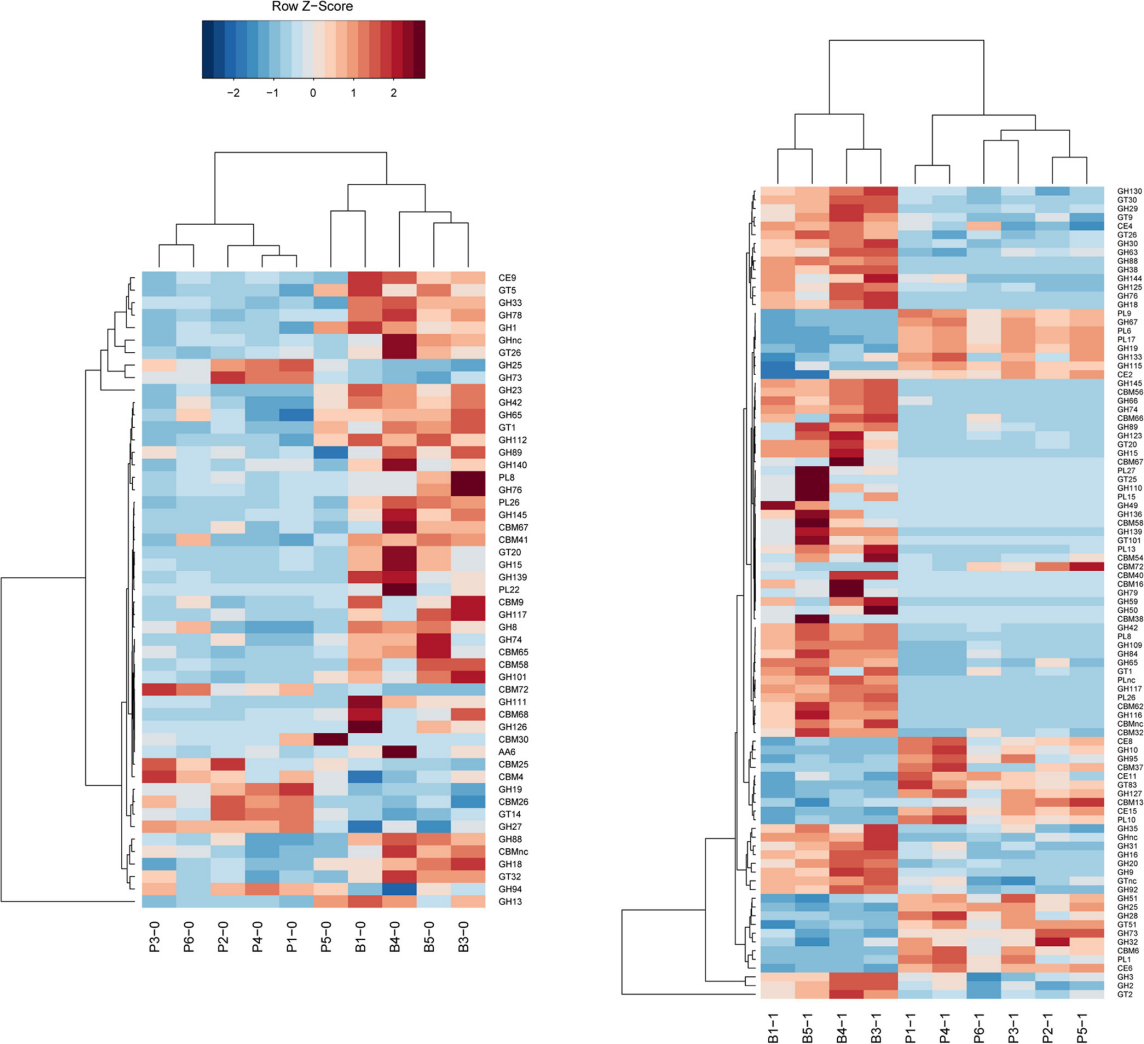
The carbohydrate-active enzymes associated with *Bacteroides* (B-) and *Prevotella* (P-) enterotypes in the original fecal samples (B0 and P0 before culturing) and after chemostat simulation (B1 and P1) were analyzed using the Carbohydrate-Active enZYmes database. The abundance of KEGG module level c annotations before and after the chemostat simulation of the two enterotypes based on the four fecal samples collected from *Bacteroides* (B-) enterotype and six collected from *Prevotella* (P-) enterotypes. The Wilcoxon *P* value was used to test the differences in abundance. *, *P* < 0.05; **, *P* < 0.01.

The carbohydrate-active enzymes were analyzed based on the CAZy database and were further compared between the two enterotype feces samples and their corresponding chemostat culture products (Fig. 7). In general, compared to the P-enterotype samples, a greater abundance of genes was observed to match carbohydrate-associated enzymes for the B-enterotype microbiota in both fecal samples and corresponding chemostat culture products. There were 23 glycoside hydrolases (GHs), 7 carbohydrate-binding modules (CBMs), 3 polysaccharide lyases (PLs), 5 glycosyltransferases (GTs), 1 carbohydrate esterase (CE), and 1 auxiliary activity (AA) enzyme identified in the metagenomic data generated from B-enterotype feces (Fig. 7). Meanwhile, only four GHs, five CBMs, and one GT were found in P-enterotype feces (Fig. 7, Table S3). Among the GHs, GH126, which is an α-amylase, was detected only in the B-enterotype feces. CBM30, which has the ability to bind to cellulose, was found only in the P-enterotype feces. With respect to the chemostat culture products, 39 GHs, 10 CBMs, 6 PLs, 9 GTs, and 1 CE were found to be associated with *Bacteroides*-dominated products. Meanwhile, 12 GHs, 5 PLs, 5 CBMs, and 5 CEs were produced by *Prevotella*-dominated chemostat products (Table S3). The chemostat culture products of P-enterotype samples exhibited higher numbers of genes encoding GH133, GH115, and GH127, which are the amylo-α-1,6-glucosidase (EC 3.2.1.33), xylan α-1,2-glucuronidase (EC 3.2.1.131), and β-l-arabinofuranosidase (EC 3.2.1.185) enzymes, respectively. The PLs associated with the *Prevotella*-dominated samples included PL1, PL6, PL9, PL10, and PL17, which are pectate lyases (EC 4.2.2.2) and alginate lyases (EC 4.2.2.3). The *Bacteroides*-dominated bacterial community produced a broader range of GHs, including GH3, GH15, GH31, GH38, GH42, and GH74, which are the α-glucosidase, glucoamylase, α-galactosidase, α-mannosidase, β-galactosidase, and endoglucanase enzymes, respectively.

Overall, the metagenomic data analysis demonstrated that the carbohydrate-active enzymes from the P-enterotype fecal samples and corresponding chemostat cultures were focused on pectate lyase and xylan hydrolysis reactions and, in particular, included amylo-α-1,6-glucosidase, which can specifically degrade IMO. In contrast, a wide range of GHs and CBMs were present in the B-enterotype fecal samples and chemostat products, which suggests that microbiota associated with the B-enterotype can live using a variety of substrates (Fig. 7).

### IMO and honey enrich *Prevotella* in mice.

Since IMO and soluble starch compose an essential substrate group for *Prevotella* and *Bacteroides* enrichment, respectively, in the *in vitro* chemostat, the next aim was to validate whether the driving effect could be observed using an animal model. To this end, a total of 48 male C57BL/6 mice (aged 4 weeks) were recruited and allotted to eight groups. Each group of mice was assigned to one of two feeding regimes (standard chow diet and high-fat diet [45%]) and four substrate groups (control, IMO, honey, and soluble starch). Honey was included as a validation of the IMO effect because among all the natural products that contain IMO, honey contains some of the highest proportions (16). As reported previously, the total oligosaccharides account for up to 16.5% of the total sugars in honey, and the majority of oligosaccharides were α-gluco-oligosaccharides, such as isomaltase-series oligosaccharides (17). The mice were fed 100 mg of substrate via oral gavage daily (except Sundays) for 7 weeks. The body weights of the mice were monitored weekly. Feces were collected at the end of the animal trial for DNA extraction and pH and moisture measurements.

The high-fat diet slightly elevated the mouse body weight with respect to those fed the standard diet, though this elevation was not statistically significant (Fig. S9a). A similar trend was also observed for the feces pH. The pH numerically increased in mice fed the high-fat diet compared to their counterparts (Fig. S9b). The substrates had no impact on either the body weight or the fecal pH. The fecal moisture content showed no differences between any of the treatments (Fig. S9c).

**FIG 8:**
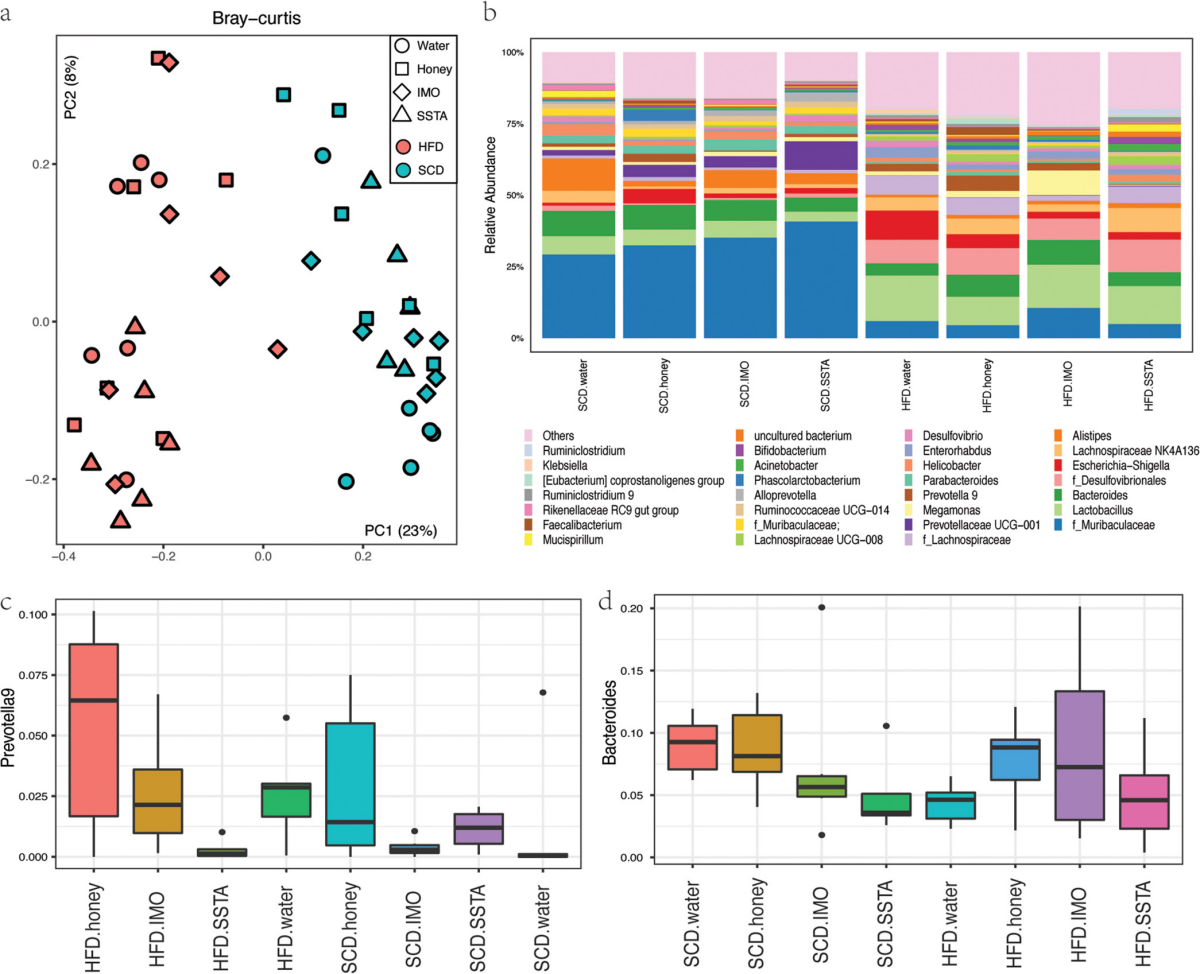
The effects of different diets and supplements on the gut microbiome and *Prevotella* and *Bacteroides* populations in a mouse model. (a) Bray-Curtis distance-based principal-component analysis (PCA) plot; (b) bacterial community compositions at the genus level and the relative abundances of (c) *Prevotella 9*; (d) *Bacteroides* in the gut microbiome (fecal samples) of mice fed different diet types. The mice were fed a high-fat diet (HFD) or standard chow diet (SCD), and these diets were supplemented with honey, isomaltooligosaccharides (IMO), soluble starch (SSTA), or water.

Diet serves as a main driver for gut microbial communities, as demonstrated by the PCA (Fig. 8a). Distinct microbial structures were observed between the mice fed the high-fat and the standard chow diets, as disclosed by the analysis of similarity (ANOSIM, *R* = 0.72 and *P* = 0.001). The substrates had no significant effects on the gut microbiota structures (ANOSIM, *R*_average_ = 0.01 and *P*_average_ = 0.35), when the groups were merged based on diet type. However, when soluble starch (SSTA) was supplemented to the high-fat diet, as opposed to the standard diet, this led to increased community diversity and richness in the mice. This was indicated by the increased number of observed OTUs (342 versus 256) and Chao1 index values (407 versus 299; Fig. S10), respectively. Significant differences between the microbial communities from these two diet groups were also observed in the PCA (ANOSIM, *R* = 0.87 and *P* = 0.001).

As expected, distinct bacterial profiles were observed at the genus level between mice fed the standard chow and high-fat diets (Fig. 8b). The gut microbiomes of the mice fed the standard diet displayed high abundances of *Muribaculaceae* family members; at least 25% of the total genera belonged to the *Muribaculaceae*, regardless of the supplement. *Bacteroides*, *Alistipes*, *Lactobacillus*, and *Prevotellaceae* UCG-001 were another five predominant genera in the standard chow diet groups. In mice fed the high-fat diet, *Lactobacillus*, *Bacteroides*, and *Lachnospiraceae* NK4A136 group were the most abundant genera. Daily treatments with IMO, SSTA, and honey barely altered the compositions of the fecal bacterial communities within each diet group, which is consistent with the PCA results.

The next step was to scrutinize the populations of specific groups of bacteria in the different treatments. *Prevotella 9* is crucial for determining a P-enterotype gut microbiome, as it is the most abundant *Prevotella*-associated taxa member. In general, the total number of *Prevotella 9* organisms in the mice was higher under the high-fat diet than under the standard chow diet (Fig. 8c; 2.8% versus 1.4%, respectively). Moreover, in the high-fat diet groups, the *Prevotella 9* abundance tended to be higher in mice that were supplied with IMO and honey than in those fed soluble starch (2.6, 0.50, and 0.2% respectively). Supplementing the standard diet with honey, which is high in IMO, also led to greater *Prevotella 9* abundance in the mice compared to the control (water) group (2.8% and 1.2%, respectively). Interestingly, the growth of *Prevotella 9* in both human and mouse feces was sustained by either IMO or IMO-rich honey. This suggests that the *Prevotella* members in both humans and mice have similar nutritional requirements (Table S4). With respect to *Bacteroides* members, none of the supplements enhanced their growth in the mouse groups fed the standard diet. Interestingly, under the high-fat diet, IMO and honey led to slight increases in *Bacteroides* abundance compared to the non-substrate-supplemented high-fat diet. Meanwhile, SSTA, which maintained a B-enterotype community in the chemostat model, had no positive impact on *Bacteroides* growth in the mouse model (Fig. 8d).

## DISCUSSION

The human gut is a very complex ecosystem that hosts trillions of bacterial cells. Diet is one of the most important external driving forces that reshapes the gut microbiota (18). In the present study, *Prevotella* was successfully enriched in *in vitro* batch and chemostat systems. Further, it was demonstrated that some substrates, such as IMO and FOS, are key nutritional resources for *Prevotella* growth. The subsequent mouse model experiment confirmed the stimulatory effect of IMO on the growth of *Prevotella*. These results provide a possible explanation for the previous results showing that the P-enterotype was highly associated with carbohydrates and simple sugars (19). Apart from the fact that higher fiber intake favors the growth of *Prevotella* in human studies (19), IMO, which are linked by mono-glucose, also support the growth of *Prevotella*.

According to the available literature, the people of the African Hadza community, who predominately possess P-enterotype gut microbiota, consume honey as a major food ingredient in daily life (20). Since honey contains high maltotriose, isomaltotriose, isomaltotetraose, and isomaltopentaose (21, 22) contents, the effects of IMO and honey on the growth of *Prevotella* were subsequently investigated in a mouse model in this study. As expected, increased *Prevotella* abundance was detected in the mice fed a high-fat diet with IMO or honey. However, only honey enhanced *Prevotella* growth when mice were fed the standard diet. The current data manifest the difficulty associated with studying the relationship between dietary ingredients and gut microbiota in *in vivo* models. Only semisynthetic diets can highlight the influence of specific dietary carbohydrate ingredients on the bacterial community. Natural ingredients, such as corn, wheat bran, and soybean cake, clearly mask and diminish the impacts of specific ingredients.

Prevotella copri is one of the predominant species present in the human colon. However, Prevotella copri strains display wide genomic diversity and can be categorized into different clades (9). Similar observations were made in the present study. The communities of the B-enterotype samples, in which more than 22 *Bacteroides* species were identified based on the metagenomics analysis, were more complex than those of the P-enterotype samples. Only four *Prevotella* species, including *P. copri*, *P. stercorea*, *P. disiens*, and *P. bivia*, were observed in the human fecal samples; *P. copri* was the most prevalent species found in all human feces. In addition, the four *Prevotella* species classified via the metagenomics analysis coincided with the OTUs obtained via 16S rRNA sequencing. The lower diversity of the *Prevotella* genus in the human samples implies that *Prevotella* may occupy a weaker evolutionary position than *Bacteroides* in the human gut. In contrast, seven and four OTUs belonging to *Prevotella* and *Prevotellaceae*, respectively, were identified in the mouse colon (Table S4). This suggests that the bacterial community in the mouse colon is more vulnerable to more complex polysaccharides than the human gut microbiota. Nevertheless, *P. copri*, which was quantitatively identified as *Prevotella 9* according to the 16S rRNA sequencing, showed a similar response to dietary IMO in both the human and mouse colon.

In any ecosystem as complex as the human gut, microbial competition for energy and carbon is intense. The efficiency of the utilization of specific carbohydrates depends on the GHs and PLs expressed by the resident microbiota (23). B-enterotype microbiota possess significantly higher numbers of genes coding for GHs, CBMs, and PLs than P-enterotype microbiota. These higher gene numbers enable the B-enterotype microbiota to utilize a wide variety of poly- and oligosaccharides. As a result, the B-enterotype has become the dominant enterotype in modern industrialized societies. In the present study, the presence of genes coding α-amylase, α-glucosidase, glucoamylase, α-galactosidase, α-mannosidase, and endoglucanase enabled the B-enterotype to successfully compete for starch when it was provided as the carbon source. In contrast, the P-enterotype microbiota showed greater expression of genes encoding pectate lyase, the enzymatic hydrolysis of xylan, and amylo-α-1,6-glucosidase. This result indicated that P-enterotype microbiota prefer to use xylan and pectin as the carbon source, which agrees with previous reports (21, 24). Interestingly, the exclusive possession of amylo-α-1,6-glucosidase by P-enterotype bacteria reveals that these bacteria favor using IMO as a growth substrate, which is in line with the results of the mouse model.

Apart from using IMO as the sole carbon source, other fermentation conditions, such as a fast dilution rate and lower pH, were also important parameters for growing P-enterotype microbiota in an *in vitro* chemostat. In fact, the dilution rate used to grow the P-enterotype microbiota was twice that used for the B-enterotype microbiota (0.04/h versus 0.08/h), and the pH was lowered to 5.5 (6.2 versus 5.5). Therefore, these factors associated with the growth of *Prevotella in vitro* may be in accordance with the colonic characteristics of people who predominantly possess P-enterotype microbiota. Support for this hypothesis comes from several studies. Liang et al. reported that people with P-enterotype gut microbiota had higher moisture, shorter intestinal transit time, and lower rates of constipation than people possessing B-enterotype microbiota (25). Su et al. found that patients with diarrhea-predominant irritable bowel syndrome (IBS-D) mainly hosted P-enterotype microbiota (26). Several microbiota landscaping studies comparing the proportions of enterotypes between rural and urban areas have linked the presence of the P-enterotype with higher SCFA production (27). Although the lower pH and higher water contents were not observed in the mice treated with IMO and honey in this study, future human clinical trials should be performed to investigate whether there is an association between IMO intake and the growth of *Prevotella* in the gut. This association could result in the control of constipation by reducing colon transit time and decreasing colonic pH.

Bifidobacteria are considered to be a beneficial bacterial group in modern Western society (28). Prebiotics, which are labeled according to their bifidogenic effect, are inferred to exert positive impacts only for B-enterotype individuals (29). Previous works have demonstrated that there is a negative correlation between the presence of *Prevotella* and *Bifidobacterium* populations in the African Hadza community. This suggests that there is a potentially negative interaction between *Prevotella* and *Bifidobacterium* (30). It is interesting that the growth of *Bifidobacterium* seems to be affected by the enterotype, because lower *Bifidobacterium* growth rates were detected for the P-enterotype fecal samples in both the *in vitro* model and the mouse model in the present study (Fig. 3c and Fig. S9d). This suggests that the growth of *Bifidobacterium* may be interfered with in the presence of *Prevotella*. This bacterial interaction within such a complex system is worthy of future study.

In conclusion, by using both an *in vitro* system and an animal model, IMO were identified as the key nutritional factor supporting the growth of P-enterotype microbiota. These results will help us to understand the nutritional requirements underlying the development of different enterotypes and will provide guidance for precisely modulating the human gut microbiome to maintain a healthy outcome.

## MATERIALS AND METHODS

### Fecal sample collection.

A total of 15 healthy human volunteers (male *n* = 8 and female *n* = 7) living in Hangzhou, China, aged between 22 and 50 years, were recruited for this study. All volunteers consumed typical Chinese food, and none claimed to be vegetarian. The donors had received no antibiotics or pro- or prebiotic treatments for at least 3 months prior to sample collection. All fecal samples were collected by natural defecation, put into a sterilized container and transported at 4°C to the laboratory within 4 h for further analysis.

### Batch culture fermentation.

Batch culture fermentation was conducted using the procedure described by Wu et al. (13). The modified YCFA growth medium contained the following (31): 10 g/L tryptone, 2.5 g/L yeast extract, 3.0 g/L 10 mg/L hemin, 1 g/L l-cysteine hydrochloride, 0.9 g/L NaCl, 0.009 g/L MgCl_2_·6H_2_O, 0.45 g/L KH_2_PO_4_, 0.45 g/L K_2_HPO_4_, 1 mg/L resazurin, 1 μg/L biotin, 1 μg/L cobalamin, 3 μg/L *p*-aminobenzoic acid, 5 μg/L folic acid, and 15 μg/L pyridoxamine. Lactulose (LAU), raffinose (RAF), fructooligosaccharides (FOS), galactooligosaccharides (GOS), IMO, mannooligosaccharides (MOS), xylooligosaccharides (XOS), inulin (INU), starch (STA), mannitol (MAI), and xylitol (XYI) were the 11 substrates that were supplemented (at 8 g/L) individually into the growth medium. Filter-sterilized mixed vitamin solution was added into the fecal slurry before inoculating it into the growth medium to give the final concentration of 0.05 mg/L thiamine and 0.05 mg/L riboflavin. The chemical structures, chain lengths, and commercial suppliers are presented in Table S1. The medium was adjusted to pH 6.5 before autoclave sterilization. Test media (5 mL) were dispensed into a 10-mL bottle sealed with butyl rubber and screw caps under anaerobic conditions.

Fresh fecal samples (0.8 g) were homogenized with 8 mL of 0.1 M anaerobic phosphate-buffered saline (pH 7.0) using an automatic fecal homogenizer (Halo Biotechnology Co., Ltd., Jiangsu, China) to make 10% (wt/vol) slurries and inoculated into culture bottles which were supplemented with 11 different MACs (FOS, GOS, IMO, XOS, RAF, MOS, INU, LAU, STA, MAI, and XYI). Fecal culture in YCFA medium without any MACs was used as a control (Y). All 180 bottles (12 media × 15 volunteers) were incubated at 37°C for 48 h, and samples were collected for further analysis.

### Single-stage chemostat fermentation.

Four paralleled single-stage chemostat systems (330 mL working volume) were set up as described previously by Lei et al. (32). The pH was automatically controlled using a pH controller (Baoxing, Shanghai, China), dilution rates were modulated by changing the peristaltic pump, and the temperature (37°C) was maintained using a circulating water bath. The physiological significance of dilution rates in the chemostat model is the retention time of food in the colon, and the pH reflects the acidic environment in the colon compartments. The pH and dilution rates needed to be set according to whether B-enterotype or P-enterotype was simulated. The systems were kept anaerobic by continuous purging with O_2_-free N_2_. Six human feces samples with an abundance of *Prevotella* were cultured individually with either IMO (six samples) or FOS (two samples) in single-stage chemostat systems. After overnight equilibration, fresh medium was supplied to the system via a peristaltic pump under a dilution rate of 0.083/h and pH = 5.5. The system was further equilibrated for at least 264 h, and then 15 mL of the chemostat culture samples was removed for analysis. In the second experiment, four fecal slurries which were classified as B-enterotype were prepared and inoculated into chemostat systems containing soluble starch. The chemostats were run under a dilution rate of 0.042/h and pH at 6.5, as described previously (14). The chemostat culture was performed for 264 h before sample collection.

### DNA extraction and 16S rRNA gene sequencing.

Bacterial genomic DNA was isolated from fecal samples and fermentation samples using a QIAamp DNA stool minikit according to the manufacturer’s instructions (Qiagen, Germantown, MD, USA). Beats were added during vortex process of DNA extraction. The DNA concentration was determined using a NanoDrop ND-2000 device (NanoDrop Technologies, USA). The DNA integrity and size were confirmed via agar gel electrophoresis (1.0%) (33), and the DNA was stored at −20°C. Bacterial 16S rRNA genes (V3-V4 region) were amplified from the extracted DNA using the barcoded primers 338F (5′-ACTCCTACGGGAGGCAGCA-3′) and 806R (5′-GGACTACHVGGGTWTCTAAT-3′). Next-generation sequencing was performed using an Illumina HiSeq 2500 system operated by Puyuan Technology Co., Ltd. (Shenzhen, China). The sequences were processed using the Quantitative Insights into Microbial Ecology (QIIME2) pipeline (34). Clean and high-quality sequences were then used for downstream analysis. The representative sequences from each operational taxonomic unit (OTU) were classified using the Ribosomal Database Project (RDP) classifier method and the SILVA database. All samples were rarefied to 5,000 reads to minimize the sequencing depth effects. Good’s coverage, alpha diversities, including the Shannon index, and richness (observed number of OTUs) were calculated using mothur (35) or QIIME2. Classification-based random forest models (default setting) were developed to identify treatment-associated bacterial members using the R program. Principal-component analysis (PCA) was evaluated for microbial distances between subjects.

### Metagenomics analysis.

The total DNA was mechanically fragmented to approximately 400 bp using Bioruptor Pico. The DNA fragments were selected and purified using magnetic beads. DNA library construction was performed following the manufacturer’s instructions (Illumina). Paired-end metagenomic sequencing was performed on the Illumina HiSeq X Ten platform (insert size of 400 bp and read length of 150 bp). High-quality reads were used for *de novo* assembly into scaffolds via IDBA-UD (version 1.1.2). Then, genes were identified from all assembled scaffolds using MetaGeneMark (version 3.26). All predicted genes were clustered at the nucleotide level using CD-HIT (version 4.5.4), and genes sharing >90% overlap and >95% identity were clustered as redundancies. The taxonomic classification of genes was performed by alignment with the National Center for Biotechnology Information (NCBI) nucleotide (NT) database using BLASTN with >70% alignment coverage of each gene. Species assignment was defined using >95% identity, genus assignment was defined using >85% identity, and phylum assignment was defined using >65% identity. The functional annotation of genes was performed according to the Kyoto Encyclopedia of Genes and Genomes (KEGG) database using BLAST KOALA (version 2). Each protein was assigned a KEGG orthologue (KO) based on the best-hit gene in the database. The carbohydrate-active enzymes associated with enterotypes were analyzed using the Carbohydrate-Active enZYmes database (CAZy, http://www.cazy.org/).

### SCFA analysis.

The concentrations of SCFAs, including acetic, propionic, isobutyric, butyric, isovaleric, and valeric acid, in the culture filtrates were determined using a gas chromatograph (GC-2010 Plus, Shimadzu, Japan) equipped with a Agilent J&W DB-FFAP column (0.32 mm by 30 m by 0.5 μm; Agilent Technologies, USA) using an H_2_ flame ionization detector. Crotonic acid (trans-2-butenoic acid) was used as an internal standard (13).

### Animals and diets.

A total of 48 male C57BL/6 mice (4 weeks old) were purchased from SLAC Laboratory Animal Co. Ltd. (China). The mice were then maintained in the animal quarters located at the animal center in Zhejiang University. After 1 week of acclimatization, the mice were divided into eight groups (six mice per group). A 2 × 4 factorial design was implemented to study the mice (two diet types and four substrate supplements). The two diet types were standard chow (25.3% crude protein, 2.6% fat, and 59.7% carbohydrate [wt/wt]; Research Diets, Shanghai SLAC Laboratory Animal Co., Ltd., China) and a high-fat diet (21.8% crude protein, 23% fat, and 41.5% carbohydrate [wt/wt]; Research Diets, Shanghai FBSH Biotechnology Co., Ltd., China; 45% of the calories were derived from fat). The reason we used both standard chow and high-fat (45%) diet regimes was due to the presence of a large amount of natural ingredients in the standard diet, which might interfere with the impacts of specific ingredients. The four substrate supplements used were IMO, honey, soluble starch (SSTA), and water (Con). The diets were made available to the mice *ad libitum*, and 100 mg of supplement was fed to each mouse daily (except on Sundays) for 7 weeks by oral gavage. The amount of honey administered was calculated to ensure that the total sugar content of the honey was 100 mg (16). The body weights and feed intake of the mice were recorded weekly. Fresh fecal samples were collected at the end of each week to analyze the pH value and moisture content. All animal work was approved by the Laboratory Animal Welfare and Ethics Committee of Zhejiang University (ethical approval no. zju2020070).

### Statistical analysis.

Nonparametric Mann-Whitney U tests (IBM SPSS Statistics version 19.0 software) were performed to analyze the differences between SCFA concentrations and Chao1 and Shannon indexes. The heatmap panel was used to display the Spearman correlation coefficients between bacteria (genus level) and SCFAs. Analysis of variance (ANOVA) followed by least significant difference (LSD) tests (IBM SPSS Statistics version 19.0 software) and Wilcoxon signed-rank tests were used to analyze the KEGG differences in the different groups.

### Data availability

All sequences were submitted to NCBI under SRA accession number SRP316642, and detailed information is presented in Table S2 in the supplemental material.
