# Supplementary material for: Isomaltooligosaccharides Sustain the Growth of Prevotella Both In Vitro and in Animal Models
Source: Microbiol Spectr. 2022 Nov 15;10(6):e02621-21. doi: 10.1128/spectrum.02621-21 (PMC9769830; doi:10.1128/spectrum.02621-21)
Supplement: Supplemental file 1 — Supplemental material. Download spectrum.02621-21-s0001.pdf, PDF file, 1.8 MB [file spectrum.02621-21-s0001.pdf]

Supplementary Information

Figure S1. Bacterial abundances at the phylum level after sample fermentation with microbiota-accessible carbohydrates (MACs). The relative abundance of the top five high-abundance phylum in feces and after fermentation. Boxes represent the interquartile range (IQR) and the line inside represents the median. Whiskers denote the lowest and highest values. The MACs tested were fructooligosaccharides (FOS), galactooligosaccharides (GOS), isomaltooligosaccharides (IMO), inulin (INU), lactulose (LAU), mannitol (MAI), mannooligosaccharides (MOS), raffinose (RAF), starch (STA), xylooligosaccharides (XOS), xylitol (XYI), and control medium (Y).

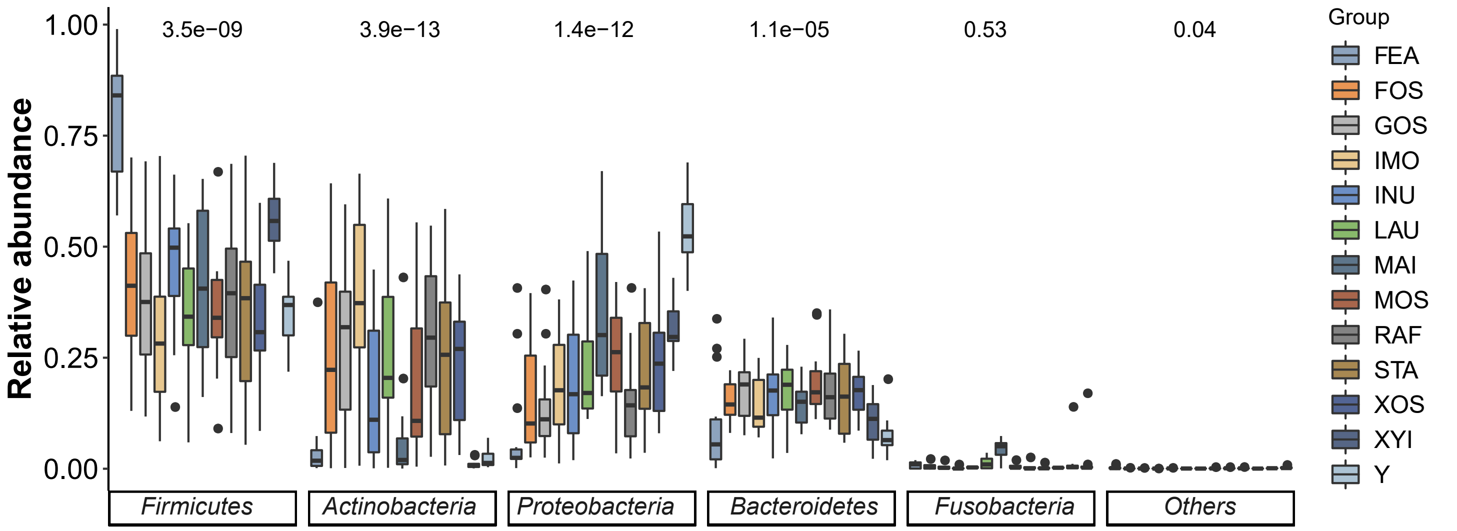

Figure S2. Genus abundance after MAC fermentation. This figure shows the relative abundance changes of genera significantly different from the original fecal sample after MACs fermentation. the size of the circles represents the average abundance of bacteria in the fermentation broth for the corresponding intervention. *p < 0.05, ***p < 0.001. Wilcoxon signed rank test (paired) was used to determine significance. The MACs tested were fructooligosaccharides (FOS), galactooligosaccharides (GOS), isomaltooligosaccharides (IMO), inulin (INU), lactulose (LAU), mannitol (MAI), mannooligosaccharides (MOS), raffinose (RAF), starch (STA), xylooligosaccharides (XOS), xylitol (XYI), and control medium (Y).

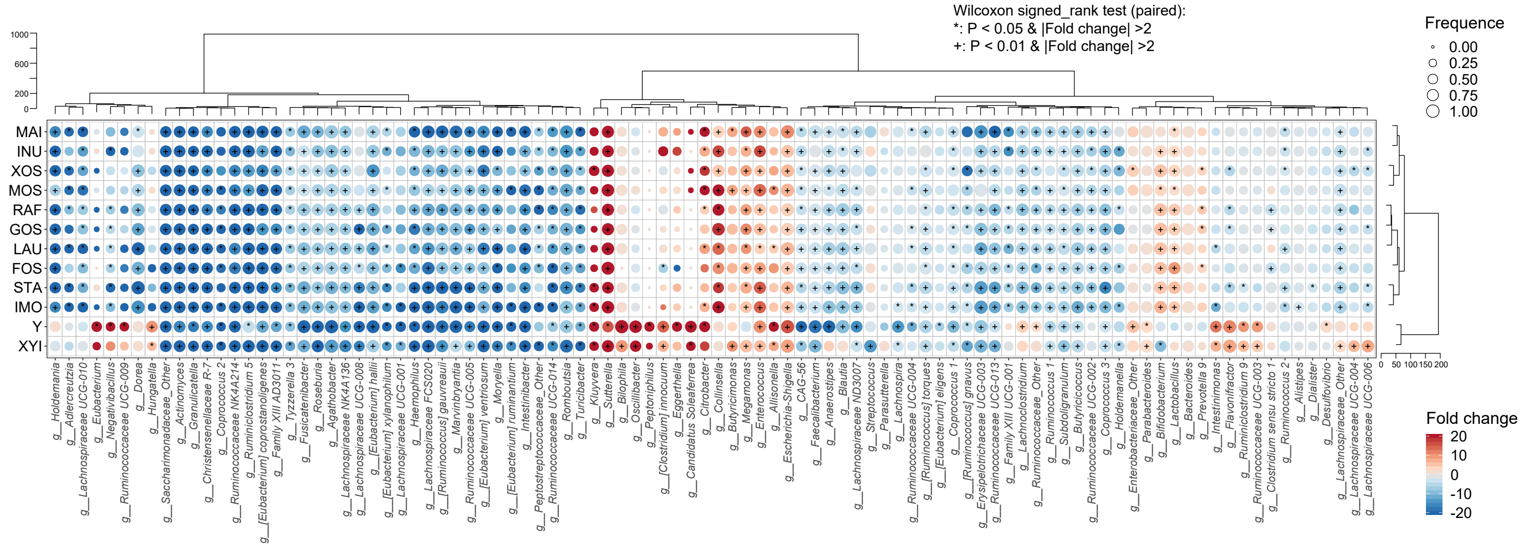

Figure S3. Validation of the Machine Learning RF Model for cluster A, B, and C, which were detected via 16S rRNA sequencing. Relative abundances of signature microbial species with different fecal structures.

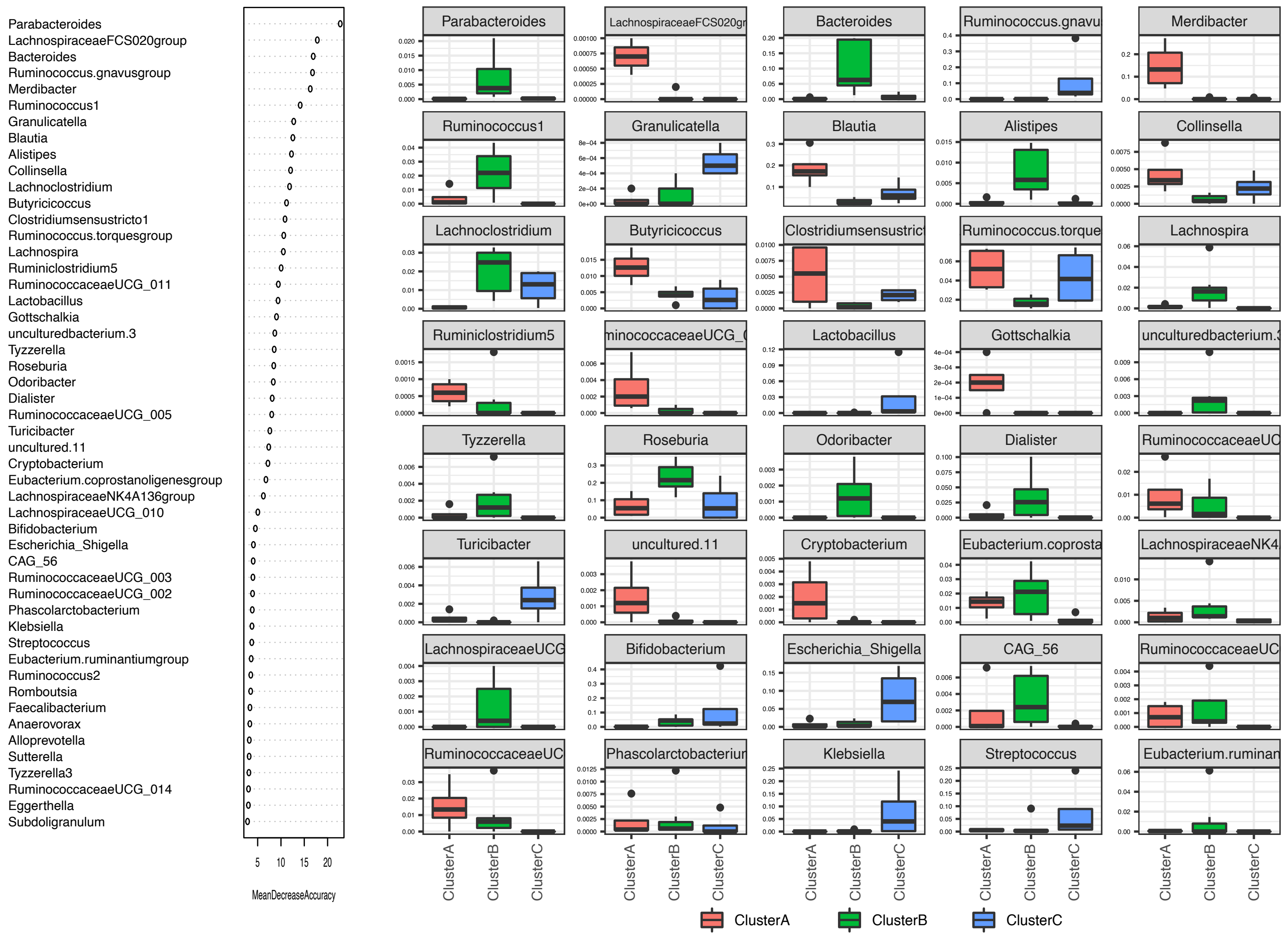

Figure S4. Comparison of short chain fatty acid (SCFA) productions in the feces (µmol/g) and after MACs fermentation (mmol/L). Each growth substrate was compared with FEA, and NS means that there is no significant difference between the medium and FEA. The MACs tested were fructooligosaccharides (FOS), galactooligosaccharides (GOS), isomaltooligosaccharides (IMO), inulin (INU), lactulose (LAU), mannitol (MAI), mannooligosaccharides (MOS), raffinose (RAF), starch (STA), xylooligosaccharides (XOS), xylitol (XYI), and control medium (Y).

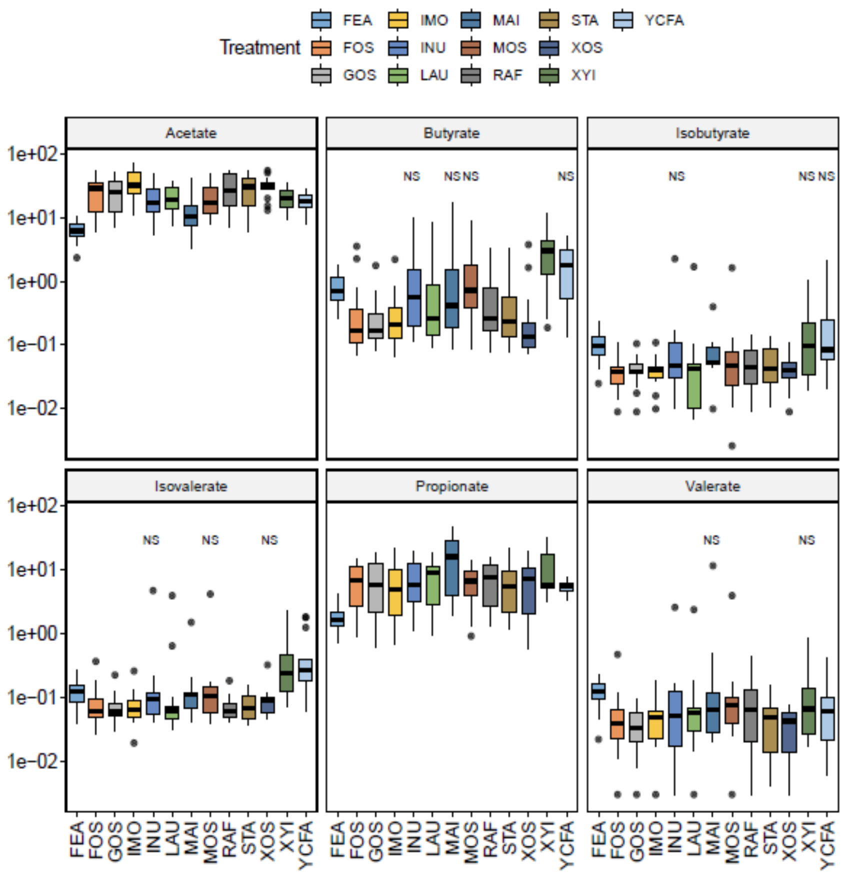

Figure S5. Correlation analysis of bacterial genera and SCFA productions. The genera presented are those of which the absolute value of correlation with MACs was greater than 0.3 and P value less than 0.05 at least in one acid. (a) acetate, (b) propionate, (c) butyrate.

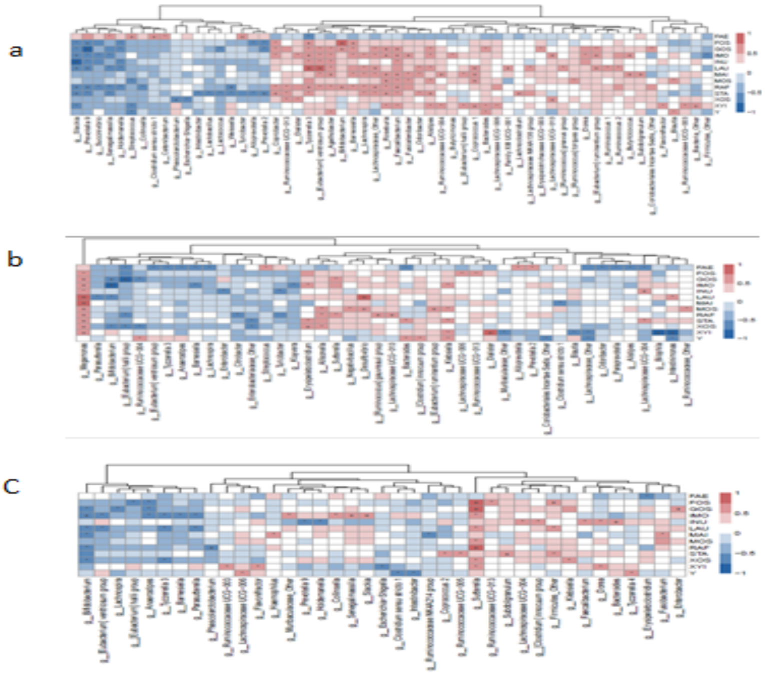

Figure S6. The effects of dilution rates on the growth of *Prevotella* in the chemostats containing IMO as the main carbon source.

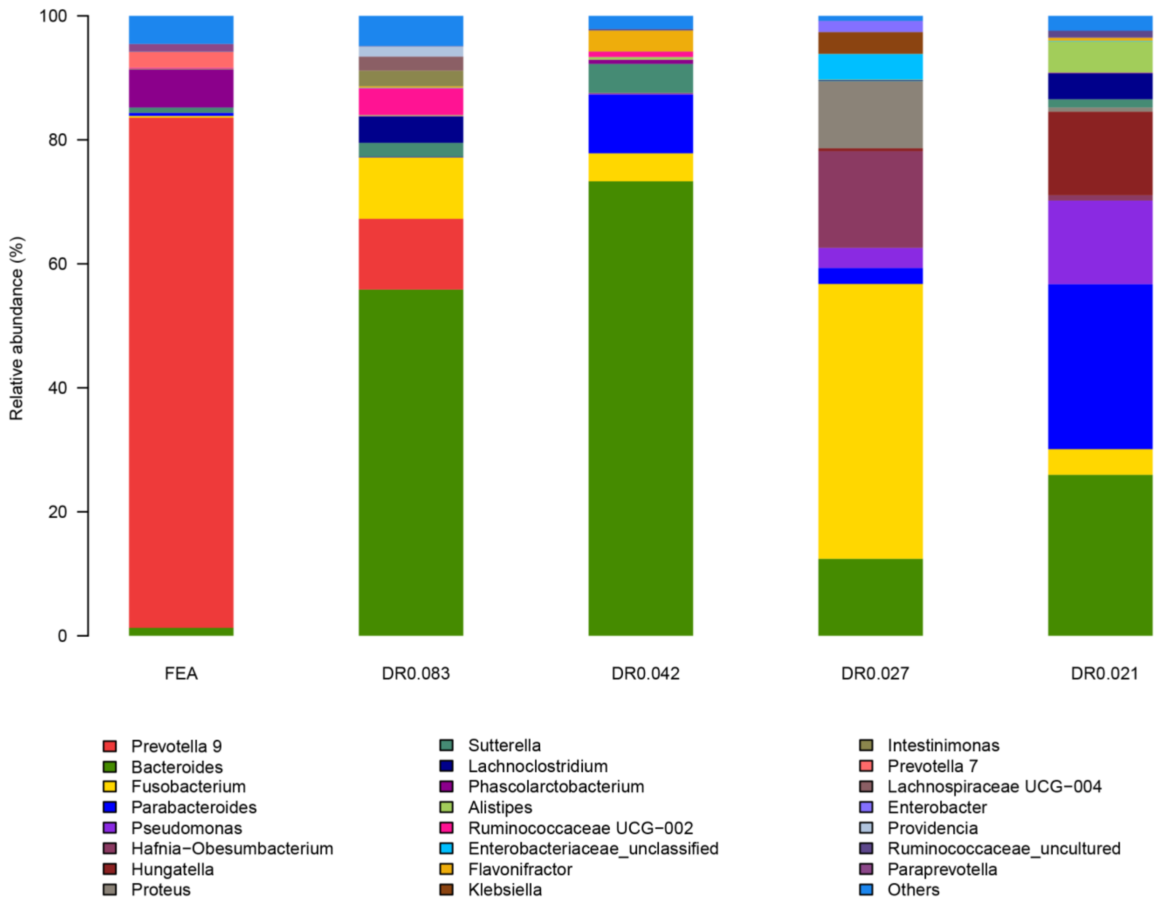

Figure S7. Effects of pH and dilution rates on the growth of *Prevotella* in the chemostates containing IMO as the main carbon source

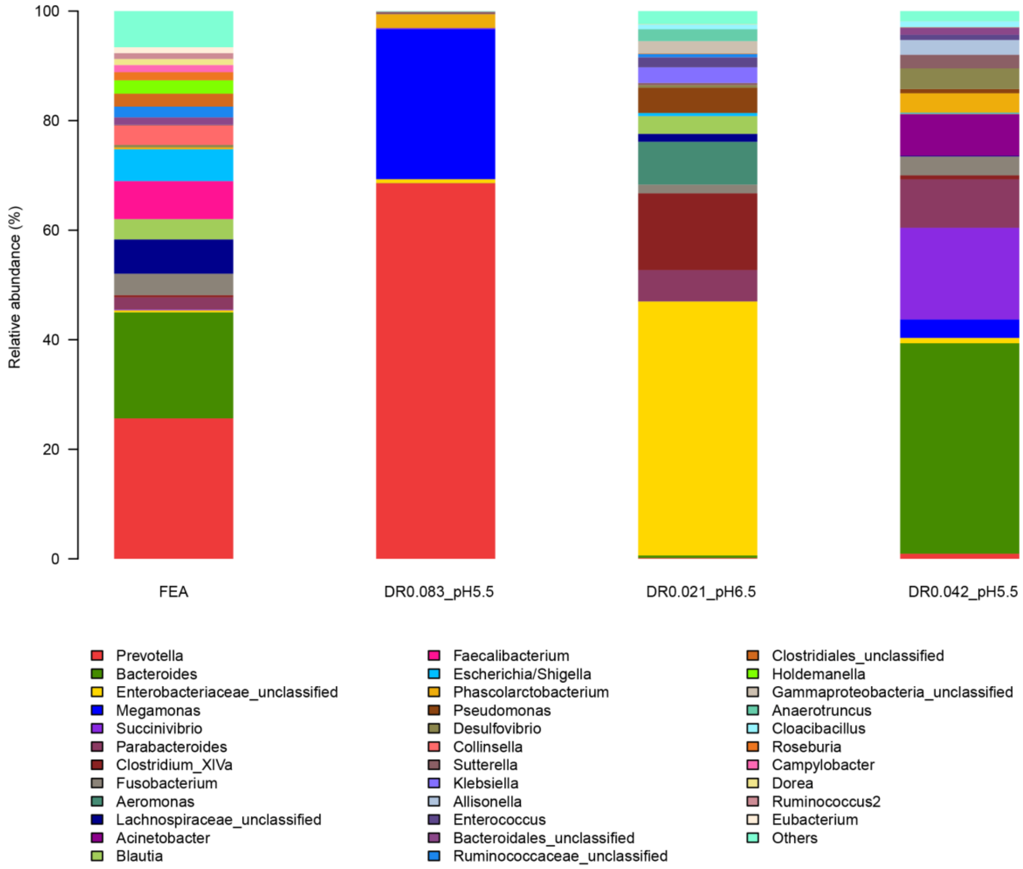

Figure S8. List of species in original fecal samples (before culturing) and chemostat culture products (after culturing) of the B- and P-enterotypes based on metagenomic analysis. This figure shows the changes in the abundance of *Bacteroides* spp. and *Prevotella* spp. of the feces and after starch and IMO fermentation. “0” means abundance before fermentation, “1” means abundance after fermentation. *Bacteroidetes* from B-enterotype, *Prevotella* from P-enterotype. The different colors of the lines represent different fecal samples.

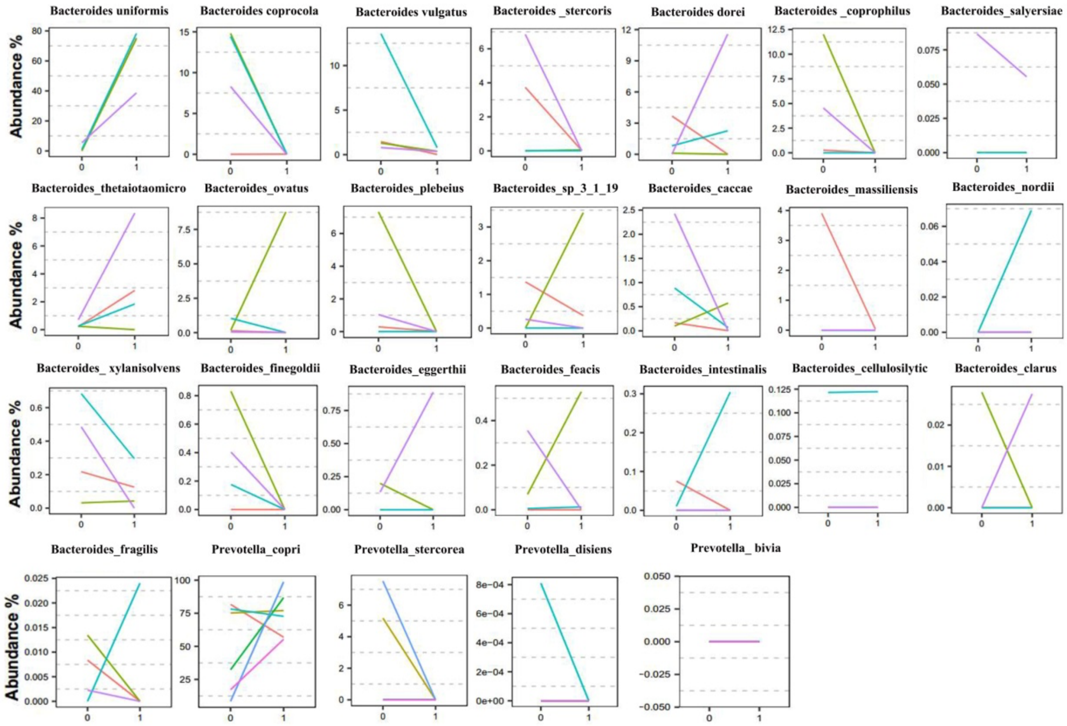
Bacteroides uniformis
Bacteroides coprocola
Bacteroides vulgatus
Bacteroides _stercoris
Bacteroides dorei
Bacteroides _coprophilus
Bacteroides_salyersiae
Bacteroides_thetaiotaomicro
Bacteroides_ovatus
Bacteroides_plebeius
Bacteroides_sp_3_1_19
Bacteroides_caccae
Bacteroides_massiliensis
Bacteroides_nordii
Bacteroides_ xylanisolvens
Bacteroides_finegoldii
Bacteroides_eggerthii
Bacteroides_feacis
Bacteroides_intestinalis
Bacteroides_cellulosilytic
Bacteroides_clarus
Bacteroides_fragilis
Prevotella_copri
Prevotella_stercorea
Prevotella_disiens
Prevotella_ bivia
Abundance %

Figure S9. Differences in metabolic date and Abundance of bifidobacteria of mice with different diet types. (a) Percentage increase in body weight of mice after 8 weeks of standard chow diet (SCD) and high fat diet (HFD) feeding compared to day 0, (b) The pH difference in feces between the two diet types, (c) The fecal moisture difference in feces between the normal and high fat diet. (d) Relative abundance of bifidobacterium in mouse feces.

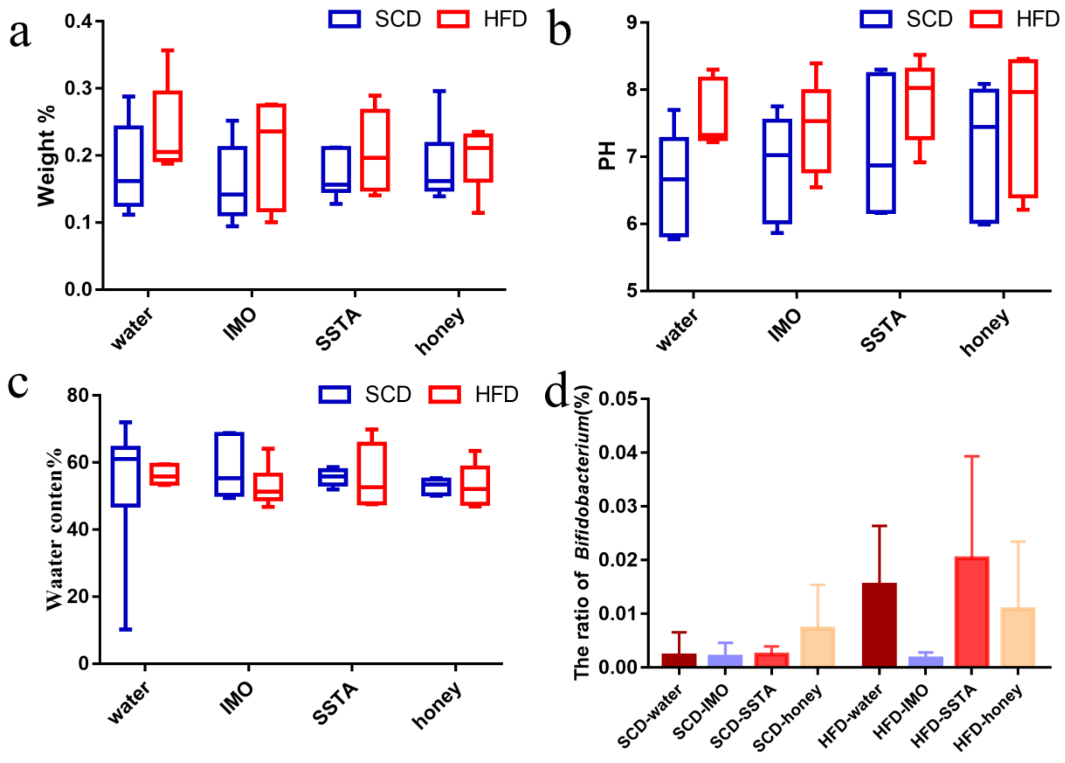

Figure S10. The alpha diversity indexes (a, observed OTUs; b, Chao1; c, Shannon; d, Simpson) of the gut microbiome in mice fed with different diet types. The mice were fed with a high-fat diet (HFD) or standard chow diet (SCD), and supplemented with honey, isomaltooligosaccharides (IMO), soluble starch (SSTA), or water.

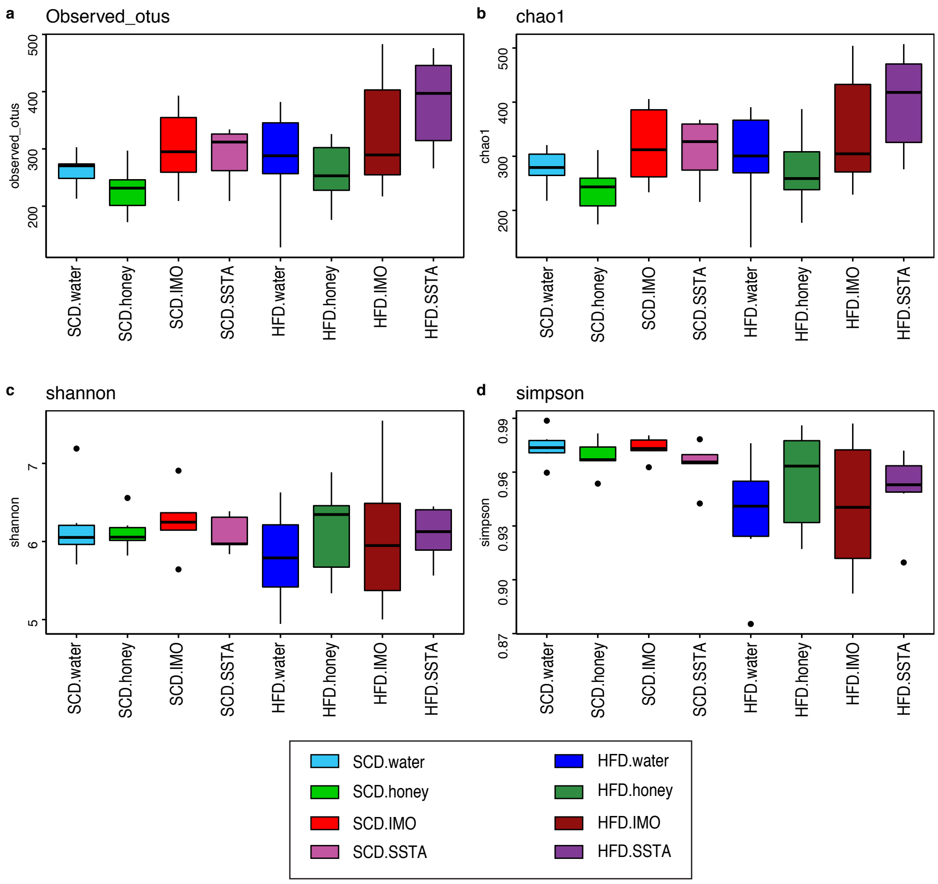

Table S1. Chemical structures and suppliers of the microbiota-accessible carbohydrates (MACs) used in this study. LAU, lactulose; RAF, raffinose; FOS, fructooligosaccharides; GOS, galactooligosaccharides; IMO, isomaltooligosaccharides; MOS, mannooligosaccharides; XOS, xylooligosaccharides; INU, inulin; STA, starch; MAI, mannitol; XYI, xylitol.

MACs	Monosaccharide composition	Glycosidic bonds	Degree of polymerization	Commercial suppliers
LAU	Galactose, fructose	β- 1, 4- glycosidic bond	2	Xiya Chemical Co. Ltd. (Shandong, China)
RAF	Galactose, fructose, glucose	α-1, 6- glycosidic bond, β-1, 2- glycosidic bond	3	Zhongtang Ruide Biotechnology Co. Ltd. (Beijing, China)
FOS	Fructose	β-2, 1- glycosidic bond	2~7	Baolingbao Biotechnology Co. Ltd. (Shandong, China)
GOS	Galactose, glucose	β-1, 4- glycosidic bond	2~6	Baolingbao Biotechnology Co. Ltd. (Shandong, China)
IMO	Glucose	α-1, 6- glycosidic bond,	5-6	Hanbang Huanyu Company (Hong Kong, China)
MOS	Mannose, glucose	β-1, 4- glycosidic bond	2~10	Yong' An Yuan He Biotechnology Co. Ltd. (Chengdu, China)
XOS	Xylose	β-1, 4- glycosidic bond	2~7	Longli Biotechnology Co. Ltd. (Shandong, China)
INU	Fructose	β-2, 1- glycosidic bond	2~60	Blingwei Fine Materials Co. Ltd. (Hebei, China)
STA	Glucose	α-1, 4- glycosidic bond	Long-chain molecules	Benchmark Chemical Reagent Co. Ltd. (Tianjin, China)
MAI	Xylose	-	Hexanehexol	Xiya Chemical Co. Ltd. (Shandong, China)
XYI	Glucose		Pentose	Xiya Chemical Co. Ltd. (Shandong, China)

Table S2. 16S rRNA gene sequencing information. FOS, fructooligosaccharides; GOS, galactooligosaccharides; IMO, isomaltooligosaccharides; INU, inulin; LAU, lactulose; MAI, mannitol; MOS, mannooligosaccharides; RAF, raffinose; STA, starch; XOS, xylooligosaccharides; XYI, xylitol; Y, control medium. OTU, operational taxonomic units.

Groups	Average raw reads	Clean reads	Observed OTUs	Shannon	PD whole tree	Chao
FEA	71556	52216	733	6.09	40.69	1519.33
FOS	51238	36554	589	5.00	40.81	1308.92
GOS	54035	38958	593	4.99	40.86	1300.84
IMO	47956	34090	457	4.33	32.69	974.62
INU	59373	42990	645	5.14	43.33	1371.13
LAU	56832	41628	613	4.97	42.94	1367.26
MAI	56832	47417	605	4.80	41.99	1357.18
MOS	56832	51142	722	5.39	47.48	1600.38
RAF	60952	44235	593	4.96	41.51	1368.67
STA	61992	44353	589	4.85	42.09	1302.99
XOS	54566	39234	639	5.18	43.37	1392.92
XYI	52504	39200	714	5.65	43.67	1509.08
Y	57056	27845	366	4.12	27.25	704.13

Table S3. Carbohydrate-active enzyme (CAZy) list, analyzed via metagenomics.

Table S3-1. Carbohydrate-associated enzymes based on the CAZy database (B0<P0). B0, P0 represented original fecal samples of Prevotella (P) and Bacteroides (B) enterotypes fecal slurries. "0" means abundance before fermentation.

ID	P0	B0	p-value	Carbohydrate-associated enzymes
GH27	0.000177	9.86E-05	0.04	α-galactosidase; α-n-acetylgalactosaminidase arabinopyranosidase
GH19	0.000134	5.35E-05	0.01	chitinase
GH25	0.00079	0.000473	0.01	lysozyme
GH73	0.000623	0.00032	0.01	lysozyme, acetylglucosaminidase specificity
GT14	0.000118	3.43E-05	0.01	β-1,3-galactosyl-o-glycosyl-glycoprotein
CBM30	4.79E-07	0	0.01	binding to cellulose for the n-terminal module
CBM26	0.000125	5.61E-05	0.02	starch-binding function demonstrated in two cases
CBM72	3.30E-06	2.37E-07	0.02	insoluble cellulose, β-1,3/1,4-mixed linked glucans, xylan, and β-mannan
CBM4	5.58E-05	2.99E-05	0.04	150 residues found in bacterial enzymes
CBM25	2.90E-05	5.07E-06	0.04	starch-binding function demonstrated in one case

Table S3-2. Carbohydrate-associated enzymes based on the CAZy database (B0>P0). B0, P0 represented original fecal samples of Prevotella (P) and Bacteroides (B) enterotypes fecal slurries. "0" means abundance before fermentation.

ID	P0	B0	p-value	Carbohydrate-associated enzymes
GH139	2.85E-06	3.12E-05	0.01	α-2-o-me-l-fucosidase
GH145	6.79E-06	4.76E-05	0.01	l-rhα-α-1,4-glca α-l-rhamnohydrolase
GH15	5.51E-06	3.01E-05	0.01	glucoamylase
GH33	0.000225	0.000423	0.01	sialidase or neuraminidase, trans-sialidase,
GH42	5.15E-05	0.00015	0.01	β-galactosidase, α-l-arabinopyranosidase
GH76	1.77E-05	0.000104	0.01	α-1,6-mannanase
GH78	0.000184	0.000412	0.01	α-l-rhamnosidase, rhamnogalacturonan α-l-rhamnohydrolase
GH88	0.000107	0.000261	0.01	d-4,5-unsaturated β-glucuronyl hydrolase
GHnc	0.000499	0.000764	0.01	-
GH111	9.37E-08	1.48E-06	0.01	-
GH101	1.26E-06	7.94E-06	0.02	endo-α-n-acetylgalactosaminidase
GH112	4.93E-05	0.000169	0.02	lacto-n-biose phosphorylase or galacto-n-biose phosphorylase
GH117	1.27E-05	5.59E-05	0.02	α-1,3-l-neoagarooligosaccharide hydrolase
GH18	0.000129	0.000303	0.02	chitinase, lysozyme
GH23	0.00068	0.001209	0.02	lysozyme type g, peptidoglycan lyase
GH8	2.46E-05	5.78E-05	0.02	chitosanase, cellulase, licheninase
GH1	0.000172	0.000402	0.04	β-glucosidase, β-galactosidase, β-mannosidase
GH13	0.00243	0.002747	0.04	α-amylase, pullulanase
GH126	0	4.18E-07	0.03	α-amylase
GH140	4.26E-05	9.00E-05	0.04	apiosidase
GH101	1.26E-06	7.94E-06	0.02	endo-α-n-acetylgalactosaminidase
GH112	4.93E-05	0.000169	0.02	lacto-n-biose phosphorylase or galacto-n-biose phosphorylase
GH117	1.27E-05	5.59E-05	0.02	α-1,3-l-neoagarooligosaccharide hydrolase
GH18	0.000129	0.000303	0.02	chitinase, lysozyme
GH23	0.00068	0.001209	0.02	lysozyme type g, peptidoglycan lyase

GH8	2.46E-05	5.78E-05	0.02	chitosanase, cellulase, licheninase
GH1	0.000172	0.000402	0.04	β-glucosidase, β-galactosidase, β-mannosidase
GH13	0.00243	0.002747	0.04	α-amylase, pullulanase
GH126	0	4.18E-07	0.03	α-amylase
GH140	4.26E-05	9.00E-05	0.04	apiosidase
GH65	6.45E-05	0.000126	0.04	α,α-trehalase, maltose phosphorylase
GH74	5.81E-06	1.88E-05	0.04	endoglucanase, xyloglucanase
GH89	7.80E-05	0.000137	0.04	α-n-acetylglucosaminidase
CBM68	9.24E-08	9.23E-07	0.04	-
CBM9	1.81E-05	5.77E-05	0.04	170 residues found so far only in xylanases
CBM41	1.33E-05	4.12E-05	0.02	modules of approx
CBM58	9.20E-07	1.23E-05	0.02	the cbm58 module of the *bacteroides thetaiotaomicron* susg protein has been shown to bind maltoheptaose
CBM65	2.49E-06	1.62E-05	0.02	cbm65a and cbm65b
CBM67	1.74E-05	3.61E-05	0.02	fujimoto
CBMnc	7.88E-05	0.000217	0.02	-
PL22	3.19E-06	2.37E-05	0.01	oligogalacturonate lyase/oligogalacturonide lyase
PL26	7.19E-06	5.46E-05	0.01	rhamnogalacturonan exolyase
PL8	4.13E-05	0.000152	0.01	hyaluronate lyase, chondroitin ac lyase, xanthan lyase
GT20	5.35E-06	3.95E-05	0.01	α,α-trehalose-phosphate synthase [udp-forming]
GT26	0.000318	0.000608	0.01	β-n-acetyl mannosaminuronyltransferase
GT1	5.89E-05	0.000159	0.02	the biosynthesis of disaccharides, oligosaccharides and polysaccharides
GT32	0.000207	0.000299	0.02	α-1,6-mannosyltransferase, α-1,4-n-acetylglucosaminyltransferase
GT5	0.00031	0.000404	0.04	glycogen glucosyltransferase, starch glucosyltransferase
CE9	0.000306	0.000497	0.01	-
AA6	6.76E-07	4.14E-06	0.04	1,4-benzoquinone reductase

Table S3-3. Carbohydrate-associated enzymes based on the CAZy database (B1<P1). B1, P1 represented chemostat culture products of Prevotella (P) and Bacteroides (B) enterotypes. “1” means abundance after fermentation.

ID	B1	P1	p-value	Carbohydrate-associated enzymes
CBM13	0.000164	0.000310	0.01	xylanase ii of *actinomadura* sp. fc7
CBM37	1.38E-05	0.000524	0.01	binding specificity to xylan, chitin, microcrystalline and phosphoric-acid swollen cellulose
CBM6	0.000195	0.001131	0.01	amorphous cellulose and β-1,4-xylan. some of these modules also bind β-1,3-glucan, β-1,3-1,4-glucan, and β-1,4-glucan
CBM72	4.00E-07	2.96E-06	0.04	-
CE15	1.69E-05	0.000465	0.01	4-o-methyl-glucuronoyl methylesterase
CE6	0.000166	0.001497	0.01	acetyl xylan esterase
CE8	0.000121	0.000749	0.01	pectin methylesterase
CE11	0.000246	0.000328	0.04	udp-3-0-acyl n-acetylglucosamine deacetylase
CE2	0.000105	0.000228	0.04	acetyl xylan esterase
GH10	5.06E-05	0.000604	0.01	endo-1,4-β-xylanase
GH115	8.05E-05	0.000215	0.01	xylan α-1,2-glucuronidase
GH127	0.000187	0.000392	0.01	β-l-arabinofuranosidase
GH19	3.62E-05	0.000212	0.01	chitinase
GH25	0.000236	0.000945	0.01	lysozyme
GH28	0.000397	0.000944	0.01	α-l-rhamnosidase, rhamnogalacturonase
GH95	0.000256	0.000650	0.01	α-l-fucosidase
GH32	0.00039	0.000739	0.01	α-l-fucosidase
GH51	0.000483	0.000804	0.01	endo-β-1,4-xylanase, β-xylosidase, α-l-arabinofuranosidase
GH67	1.44E-05	0.000195	0.01	copper-dependent lytic polysaccharide monooxygenases
GH73	0.000334	0.000862	0.01	mannosyl-glycoprotein endo-β-n-acetylglucosaminidase
GH133	0.000149	0.000202	0.04	amylo-α-1,6-glucosidase
GT51	0.000617	0.001053	0.01	murein polymerase

GT83	0.000182	0.000290	0.01	4-amino-4-deoxy-β-l-arabinosyltransferase.
PL1	8.96E-05	0.001062	0.01	pectate lyase
PL10	4.43E-05	0.000351	0.01	pectate lyase
PL17	7.91E-06	0.000185	0.01	alginate lyase
PL6	6.82E-06	0.000187	0.01	alginate lyase, chondroitinase b
PL9	1.67E-05	0.000209	0.01	pectate lyase, exopolygalacturonate lyase

Table S3-4. Carbohydrate-associated enzymes based on the CAZy database (B1>P1). B1, P1 represented chemostat culture products of Prevotella (P) and Bacteroides (B) enterotypes. “1” means abundance after fermentation.

ID	B1	P1	p-value	Carbohydrate-associated enzymes
CE4	0.000594	0.000438	0.01	acetyl xylan esterase, chitin deacetylase
CBM58	1.21E-05	2.32E-08	0.01	the cbm58 module of the *bacteroides thetaiotaomicron* susg protein has been shown to bind with maltoheptaose
CBM66	0.000117	1.18E-05	0.02	the cbm66 module, derived from the *bacillus subtilis* exo-acting β-fructosidase sacc, targets the terminal fructoside residue of fructans
CBM16	6.83E-07	0	0.03	carbohydrate binding module 16 binding to cellulose and glucomannan demonstrated
CBM32	0.000304	6.11E-05	0.01	binding to polygalacturonic acid has been shown for a yersinia member (pmid: 17292916). binding to lacnac (β-d-galactosyl-1,4-β-d-n-acetylglucosamine) has been shown for an n-acetylglucosaminidase from *clostridium perfringens* (pmid: 16990278)
CBM38	6.46E-05	6.08E-08	0.01	the inulin-binding function has been demonstrated in the case of the cycloinulooligosaccharide fructanotransferase from *paenibacillus macerans* (*bacillus macerans*)
CBM56	0.000149	3.45E-06	0.01	β-1,3-glucan binding function demonstrated
CBM62	0.000359	3.14E-06	0.01	the cbm62 module of *clostridium thermocellum* cthe_2193 protein binds galactose moieties found on xyloglucan, arabinogalactan, and galactomannan
CBM67	6.58E-05	5.39E-07	0.01	l-rhamnose binding activity
CBM40	2.89E-06	2.43E-08	0.03	sialic acid binding function
CBMnc	0.000337	5.95E-06	0.01	-
GH109	0.000198	1.74E-05	0.01	α-n-acetylgalactosaminidase
GH110	3.48E-05	1.86E-06	0.01	α-galactosidase
GH116	0.000321	1.55E-07	0.01	β-glucosidase, β-xylosidase
GH117	0.000279	4.42E-07	0.01	α-1,3-l-neoagarooligosaccharide hydrolase
GH123	0.000101	4.02E-06	0.01	β-n-acetylgalactosaminidase
GH125	0.000676	0.000140	0.01	exo-α-1,6-mannosidase
GH130	0.000744	0.000404	0.01	β-1,4-mannooligosaccharide phosphorylase
GH136	1.66E-05	1.39E-07	0.01	lacto-n-biosidase
GH139	1.50E-05	5.58E-08	0.01	α-2-o-me-l-fucosidase

GH145	0.000166	2.89E-07	0.01	l-rhα-α-1,4-glca α-l-rhamnohydrolase
GH15	9.24E-05	1.25E-06	0.01	glucoamylase
GH16	0.001051	0.000402	0.01	xyloglucan:xyloglucosyltransferase, keratan-sulfate endo-1,4-β-galactosidase
GH18	0.000854	5.58E-05	0.01	chitinase, lysozyme, endo-β-n-acetylglucosaminidase
GH20	0.001188	0.000283	0.01	β-hexosaminidase, lacto-n-biosidase
GH29	0.000749	0.000254	0.01	α-l-fucosidase, α-1,3/1,4-l-fucosidase
GH3	0.004068	0.002415	0.01	β-glucosidase, xylan 1,4-β-xylosidase
GH30	0.000416	0.000166	0.01	endo-β-1,4-xylanase, β-glucosidase, β-glucuronidase
GH31	0.000927	0.000494	0.01	α-glucosidase, α-galactosidase
GH38	0.000459	1.62E-05	0.01	α-mannosidase, mannosyl-oligosaccharide α-1,2-mannosidase.
GH42	0.000205	1.05E-05	0.01	β-galactosidase, α-l-arabinopyranosidase
GH63	0.000388	0.000111	0.01	α-glucosidase, α-1,3-glucosidase, α-glucosidase
GH65	0.000184	2.61E-05	0.01	α,α-trehalase, maltose phosphorylase
GH66	0.000126	7.35E-06	0.01	cycloisomaltooligosaccharide glucanotransferase, dextranase
GH74	0.000118	8.16E-08	0.01	endoglucanase, oligoxyloglucan reducing end-specific cellobiohydrolase
GH76	0.000588	7.16E-07	0.01	α-1,6-mannanase
GH84	0.000167	1.73E-05	0.01	n-acetyl β-glucosaminidase, hyaluronidase
GH88	0.000591	1.91E-05	0.01	d-4,5-unsaturated β-glucuronyl hydrolase
GH9	0.001131	6.59E-05	0.01	endoglucanase, endo-β-1,3(4)-glucanase.
GH92	0.001749	0.000474	0.01	mannosyl-oligosaccharide α-1,2-mannosidase
GH144	0.00055	8.84E-05	0.02	endo-β-1,2-glucanase
GH2	0.003466	0.002241	0.02	β-galactosidase, β-mannosidase, β-glucuronidase
GH35	0.001191	0.000569	0.02	β-galactosidase, exo-β-glucosaminidase
GH89	9.70E-05	1.17E-05	0.02	α-n-acetylglucosaminidase
GH59	2.62E-08	0	0.03	β-galactosidase, galactocerebrosidase
GH79	2.46E-07	0	0.03	β-glucuronidase, hyaluronoglucuronidase
GH49	3.47E-05	1.64E-06	0.04	dextranase
GH50	2.07E-05	2.02E-07	0.04	β-agarase
GHnc	0.001153	0.000502	0.01	-
GT2	0.009919	0.007651	0.01	cellulose synthase, chitin synthase

GT20	0.000104	5.98E-06	0.01	α,α-trehalose-phosphate synthase [udp-forming], glucosylglycerol-phosphate synthase
GT26	0.000689	0.000184	0.01	β-n-acetyl mannosaminuronyltransferase
GT30	0.000713	0.000332	0.01	α-3-deoxy-d-manno-octulosonic-acid (kdo) transferase
GT9	0.000658	0.000452	0.01	lipopolysaccharide n-acetylglucosaminyltransferase, heptosyltransferase
GT101	1.85E-05	5.73E-08	0.01	glucosyltransferase
GT25	2.37E-05	2.09E-07	0.04	lipopolysaccharide β-1,4-galactosyltransferase
GT1	0.000175	3.29E-05	0.04	udp-glucuronosyltransferase
GTnc	0.00157	0.000633	0.01	-
PL15	4.42E-05	3.07E-07	0.01	oligo-alginate lyase
PL26	0.000229	3.76E-07	0.01	rhamnogalacturonan exolyase
PL27	3.21E-05	3.05E-07	0.01	l-rhamnose-α-1,4-d-glucuronate lyase
PL8	0.000204	3.81E-06	0.01	hyaluronate lyase, chondroitin ac lyase
PLnc	0.000273	7.68E-06	0.01	-
PL13	9.97E-06	2.27E-07	0.01	heparin lyase

Table S4. The average relative abundance of *prevotella* in mouse feces and chemostat culture products based on 16s analysis.

Table S4-a. The average relative abundance of prevotella in mice feces. SCD means standard chow diet group, HFD means high fat diet group. The sample size of each group was six.

GROUP	SCD-water	SCD-IMO	SCD-SSTA	SCD-honey	HFD-water	HFD-IMO	HFD-SSTA	HFD-honey
Prevotella	1E-05	6E-05	0.0002	4E-06	0.0004	0.000368	6.22E-05	0.00023
Prevotella 1	1E-05	8E-05	0.0002	0.0062	6E-05	0.00011	0.000273	3.13E-05
Prevotella 2	0.0009	0.0004	0.0007	0.0063	0.0029	0.002925	0.000296	0.011064
Prevotella 7	0.0002	6E-05	0	0	0.0003	0.000973	0.000163	0.000352
Prevotella 9	0.011	0.0049	0.0109	0.0253	0.029	0.042577	0.004057	0.07767
Prevotellaceae Ga6A1 group	1E-05	8E-05	2E-05	0.0001	0.0001	0	0.000317	0.000176
Prevotellaceae NK3B31 group	6E-05	1E-05	0.0002	1E-05	0.0004	0.000631	0	3.17E-06
Prevotellaceae UCG-001	0.0161	0.0343	0.0674	0.0311	0.0005	0.001804	0.002542	0.000859
Prevotellaceae UCG-003	0	5E-05	0	0	4E-05	4.1E-06	0	1.94E-05
Prevotellaceae UCG-004	3E-05	9E-06	0	0.0002	5E-06	6.25E-05	9.81E-05	4.25E-05

Table S4-b. The average relative abundance of prevotella in original fecal samples (before culturing) and chemostat culture products (after culturing) of the B- and P-enterotypes based on 16s analysis. IMO, SSTA and FOS were different carbon source groups , “N” represents the number of samples from each group.

GROUP	IMO-Faeces N=6P-	IMO-Fermentation N=6P-	SSTA-Faeces N=4B-	SSTA-Fermentation N=4B-	FOS-Faeces N=2P-	FOS-Fermentation N=2P-
Prevotella	0	0	0	0	0	0
Prevotella 1	0	0	0	0	9.42E-05	0.000627
Prevotella 2	0.07607	0.006967	0	0	0.00945	0.019996
Prevotella 7	0.00669	4.89E-05	0	1.18E-04	0.00049	0.001571
Prevotella 9	0.43717	0.598298	0.01766	9.11E-06	0.33589	0.094187
Prevotellaceae Ga6A1 group	0	0	3.6E-05	0.000227629	0	0
Prevotellaceae NK3B31 group	0	0	1.8E-05	9.10515E-06	0	0
Prevotellaceae UCG-001	0	0	0	0.000209418	0	0
Prevotellaceae UCG-003	0	0	9.1E-06	3.64206E-05	0	0
Prevotellaceae UCG-004	0	0	0	0	0	0
